# Induction of RAC1 protein translation and MKK7/JNK-dependent autophagy through dicer/miR-145/SOX2/miR-365a axis contributes to isorhapontigenin (ISO) inhibition of human bladder cancer invasion

**DOI:** 10.1038/s41419-022-05205-w

**Published:** 2022-08-31

**Authors:** Xiaohui Hua, Daimin Xiang, Mengxin Guo, Xiaohui Qian, Ruifan Chen, Tengda Li, Zhongxian Tian, Jiheng Xu, Chao Huang, Qipeng Xie, Chuanshu Huang

**Affiliations:** 1 Oujiang Laboratory (Zhejiang Lab for Regenerative Medicine, Vision and Brain Health), School of Laboratory Medicine and Life Sciences, Wenzhou Medical University, Wenzhou, Zhejiang 325035 China; 2 Department of Occupational Health and Environmental Health, School of Public Health, Anhui Medical University, Hefei, Anhui 230032 China; 3 State Key Laboratory of Oncogenes and Related Genes, Shanghai Cancer Institute, Renji Hospital, Shanghai Jiao Tong University School of Medicine, Shanghai, 200127 China; 4 Department of Urology, Union Hospital, Tongji Medical College, Huazhong University of Science and Technology, Wuhan, 430022 China

**Keywords:** Cancer therapy, Autophagy

## Abstract

Although our previous studies have identified that isorhapontigenin (ISO) is able to initiate autophagy in human bladder cancer (BC) cells by activating JNK/C-Jun/SESN2 axis and possesses an inhibitory effect on BC cell growth, association of autophagy directly with inhibition of BC invasion has never been explored. Also, upstream cascade responsible for ISO activating JNK remains unknown. Thus, we explored both important questions in the current study and discovered that ISO treatment initiated RAC1 protein translation, and its downstream kinase MKK7/JNK phosphorylation/activation, and in turn promoted autophagic responses in human BC cells. Inhibition of autophagy abolished ISO inhibition of BC invasion, revealing that autophagy inhibition was crucial for ISO inhibition of BC invasion. Consistently, knockout of *RAC1* also attenuated induction of autophagy and inhibition of BC invasion by ISO treatment. Mechanistic studies showed that upregulation of RAC1 translation was due to ISO inhibition of miR-365a transcription, which reduced miR-365a binding to the 3’-UTR of *RAC1* mRNA. Further study indicated that inhibition of miR-365a transcription was caused by downregulation of its transcription factor SOX2, while ISO-promoted Dicer protein translation increased miR-145 maturation, and consequently downregulating SOX2 expression. These findings not only provide a novel insight into the understanding association of autophagy induction with BC invasion inhibition by ISO, but also identify an upstream regulatory cascade, Dicer/miR145/SOX2/miR365a/RAC1, leading to MKK7/JNKs activation and autophagy induction.

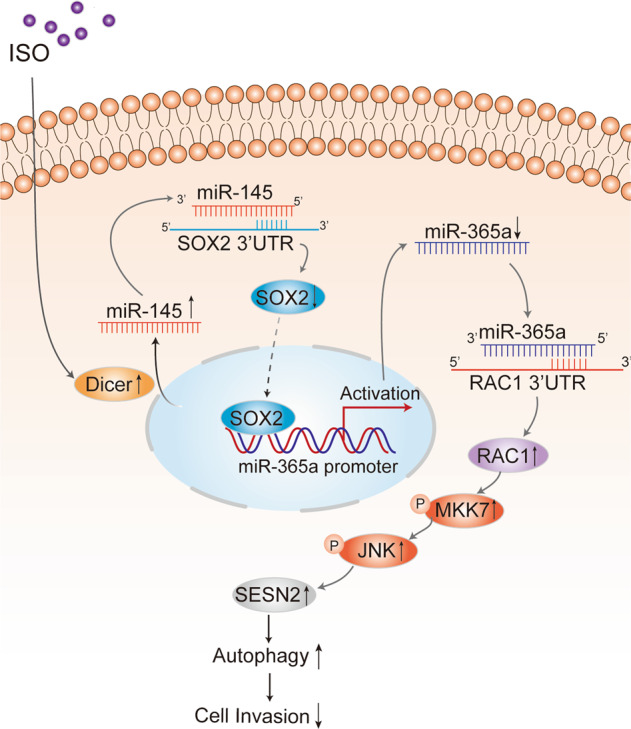

## Introduction

Bladder cancer (BC) ranks as the fourth most frequently diagnosed cancer in men and is also a common malignancy in women worldwide [1]. Because high-grade invasive bladder cancers can progress to life-threatening metastases and are responsible for almost 100% of death from this disease [2, 3], identifying a natural compound that specifically inhibits BC invasion and metastasis is tremendously important for potentially reducing mortality as a result of this disease.

The Chinese herb Gnetum cleistostachyum has been used as an anticancer agent for thousands of years [4]. Isorhapontigenin (ISO) is a new derivative of stilbene compound that was originally isolated from this Chinese herb [5] and also exists in the grape and its products as the main dietary source [6]. ISO, as a new resveratrol analog, has stronger potency and better pharmacokinetics, with IC_50_ values at least two-fold lower than those of resveratrol [7]. In our published studies, we have established the multiple anti-cancer activities of ISO in human BC cells [8–10]. Though low doses (5-10 µM) of ISO effectively induce G0/G1 growth arrest through inhibition of cyclin D1 expression [2], relative higher doses (20-60 µM) induce apoptotic responses in cancer cells *via* inhibition of XIAP gene transcription [4]. Our studies have demonstrated that in vivo treatment with ISO at 150 mg/kg/day markedly inhibits over 90% of muscle-invasive BC (MIBC) formation in BBN-exposed mice ^5^. Our studies have also showed that ISO treatment impairs anchorage-independent growth of human invasive BC cells concurrently with induction of autophagy [8, 9, 11]. Moreover, our previous publications have consistently shown that ISO treatment has a significant inhibitory effect on cell invasion, with no significant effect on cell migration [8, 9, 11]. Collectively, these findings suggest that ISO may be a promising agent for inhibition of MIBC formation and progression and provide a strong impetus for the further investigation of the association of autophagy induction with the inhibitory effects of ISO on MIBC invasion and formation. Thus, the association of ISO inhibition of invasion with autophagic responses in human high invasive BC cells as well as the molecular mechanisms underlying its biological effects have been the main focus of the current studies.

MicroRNAs (miRNAs) are 18–25 nucleotide non-coding RNAs that can bind to target mRNAs, usually in their 3′-untranslated regions (UTR), to inhibit their expression by either inducing their degradation or repressing their translation [12]. miRNAs have emerged as integral components of nearly every biological process, including cell proliferation, migration, invasion, differentiation, apoptosis, and angiogenesis [13]. miR-145, one of the first predicted based on its homology to a verified miRNA from mouse [14] and subsequently verified in humans [15], was downregulated in many types of cancers, suggesting that it may serve as a tumor suppressor [16, 17]. Our most recent studies demonstrate that ISO treatment results in an increased miR-145 level in human invasive BC cells [18]. However, the upstream regulators and its downstream effectors, as well as its function in ISO anti-cancer effect, have not explored yet. miR-365 has been reported to play roles in tumor progression and development in several types of human cancers, including lung cancer [19], osteosarcoma [20], and colon cancer [21]. Although miR-365 was found to be upregulated and function as an oncogene in gastric cancer [22], oral squamous cell carcinoma [23] and cutaneous squamous cell carcinoma [24], the biological function and underlying molecular mechanisms of miR-365a in human BCs has never been elucidated yet. In the current studies, we found that both miR-145 and miR-365a were consequently involved in ISO activation of RAC1/MKK7/JNKs axis-dependent autophagy and its mediated inhibition of BC invasion.

Small GTPase 1 (RAC1) is a small (~21 kDa) signaling G protein and a member of the Rac subfamily of the family Rho family of GTPases and has ubiquitous tissue expression [25]. As a modulator of the cytoskeleton, RAC1 activity is essential for many normal cellular activities [26]. RAC1 appears to be dysregulated in both expression and activity in a variety of tumors, which correlates well with aggressive growth and other malignant characteristics, including tumorigenesis, invasion, and metastasis [27, 28]. As such, this protein may act as an effective target for drugs aimed at disrupting these malignancies. In addition, many upstream factors, including miRNAs, have been reported to regulate RAC1. For example, miR-142 could directly target and negatively regulates RAC1 to suppress hepatocellular carcinoma cell migration and invasion [29]; miR-101 acts as a novel suppressor by targeting RAC1 and inhibits the migration and invasion in thyroid cancer cells [30]. In the current study, we found that ISO-inhibited miR-365a expression results in the reduction of miR-365a binding to RAC1 mRNA 3′-UTR, subsequently increasing RAC1 protein translation, thereby activating MKK7/JNK-dependent autophagic cell invasion inhibition in human BC cells.

## Materials and Methods

### Reagents and Plasmids

Protein synthesis inhibitor cycloheximide (CHX) was bought from Calbiochem (San Diego, CA, USA). The dual luciferase assay kit, TRIzol reagent and SuperScriptTM First-Strand Synthesis system were obtained from Promega (Madison, WI, USA) and Invitrogen (Grand Island, NY, USA), respectively. PolyJet^TM^ DNA in Vitro Transfection Reagent was purchased from SignaGen Laboratories (Rockville, MD, USA). The plasmids of CRISPR/Cas9 RAC1 and its control vector were bought from Open Biosystems (Thermo Fisher Scientific, Pittsburgh, PA, USA). miR-145 promoter luciferase plasmid, miR-365a promoter luciferase plasmid, RAC1 3′UTR luciferase plasmid, Dicer 3’UTR luciferase plasmid, miR-365a overexpression plasmid were constructed by us. A fragment spanning 1228 bp relative to the mRNA 3’UTR site of human *RAC1* genomic sequence was produced by PCR with the forward primer 5′- CCG CTC GAG CTT CGC ACT CAA TGC CAA CT -3′ and the reverse primer 5′- TCC GAG CTC GAC CCA AAG GAA CAT CAA TAG G -3′. The PCR products were subcloned into the XhoI and SacI sites of pMIR-Report vector (Promega Co., E1751) to generate the RAC1 3′UTR luciferase plasmid. The construct was confirmed by DNA sequencing (GENEWIZ). All transfectants were used as a mass pool culture rather than individual clones.

### Cell lines and Cell Culture

UMUC3 cells were maintained at 37°C in 5% CO_2_ incubator with Dulbecco’s modified Eagle’s medium (DMEM) supplemented with 10% fetal bovine serum (FBS), 2 mM L-glutamine, and 25 µg/ml of gentamicin [9]. The cultures were dissociated with 0.5% trypsin and transferred to new six-well plate twice a week. FBS was purchased from Nova-Tech (Grand Island, NE). ISO with over 99% purity was purchased by Higher Biotech (Shanghai, China) and dissolved in dimethyl sulfoxide (DMSO, Sigma) to make a stock concentration at 20 mM and the same concentration (0.1%, v/v) of DMSO was used as a vehicle control in all experiments.

### Western blot

Western blot assay was tested as previously described [31, 32]. Briefly, cells were plated in six-well plates and cultured in normal FBS (10% for UMUC3 cells) medium at 70–80% confluence. The cells were then cultured in 0.1% FBS medium for 12 h and subsequently treated with different doses of ISO for the indicated time. The cells were washed once with ice-cold phosphate-buffered saline (PBS), and cell lysates were prepared with a lysis buffer [10 mM Tris–HCl (pH 7.4), 1% SDS and 1 mM Na3VO4]. An equal amount (100 µg) of total protein from each cell lysate was subjected to Western blot with the indicated primary antibodies. Immunoreactive bands were detected by using the alkaline phosphatase-linked secondary antibody and the ECF Western blotting system (Amersham Biosciences, Piscataway, NJ). The images were acquired, and the protein levels were quantified by using the Typhoon FLA 7000 imager (GE Healthcare, Pittsburgh, PA).

### RT-PCR and quantitative RT-PCR

Total RNA was extracted with TRIzol reagent (Invitrogen Corp., Carlsbad, CA), and cDNAs were synthesized by using the SuperScript® IV First-Strand Synthesis System for RT-PCR (Invitrogen Corp.). A pair of oligonucleotides (forward: 5′-AGA AGG CTG GGG CTC ATT TG -3′ and reverse: 5′-AGG GGC CAT CCA CAG TCT TC -3′) were adopted to amplify human *GAPDH* cDNA as a loading control. The human *RAC1* specific PCR primers (forward: 5′- ATC AAG TGT GTG GTG GTG GG -3′; reverse: 5′-CCA GCT GTA TCC CAT AAG CCC A -3′) The human *Dicer* specific PCR primers (forward: 5′-GAG TGT TTG AGG GAT AG -3′; reverse: 5′-CTG AGG TAT GGG TTT GG-3′). The PCR products were then separated on 2% agarose gels and stained with ethidium bromide. The images were visualized and scanned by using the UV lights with a FluorChem SP imaging system (Alpha Innotech, San Leandro, CA) as previously described [32]. The Quantitative RT-PCR analysis was conducted for miRNA assay. Total RNA was extracted using the miRNeasy Mini Kit (Qiagen, Valencia, CA), and analysis of miRNAs expression was performed by the miScript PCR system (Qiagen) and the 7900HT Fast Real-time PCR system. The ΔΔCT value was used to calculate the relative expression of miRNAs, using U6 as endogenous control.

### In vitro cell migration and invasion assays

In vitro migration and invasion assays were conducted by using Transwell chambers (for migration assay) or Transwell precoated Matrigel chambers (for invasion assay) according to the manufacturer’s protocol (BD Biosciences) as described previously [33]. Briefly, 700 µL of medium containing 10% FBS (for UMUC3) with or without 10 µM of ISO was added to the lower chambers, whereas homogeneous single-cell suspensions (5 × 10^4^ cells/well) in 0.1% FBS medium with or without 10 µM of ISO as indicated were added to the upper chambers. The Transwell plates were incubated in 5% CO2 incubator at 37 °C for 24 h, and thereafter were washed with 1×PBS, fixed with 3.7% formaldehyde for 5 min, washed twice, re-fixed with 100% methanol for 20 min, washed twice again, and then stained with Giemsa (1:10 in PBS) for 30 min in the dark. The non-migration or non-invading cells were scraped off on the top of the chamber. The migration and invasion rates were quantified by counting the migration and invaded cells at least three random fields under a light microscope (Olympus).

### Luciferase assay

As described in our previous studies [2, 4], a dual-luciferase reporter assay was performed by using the luciferase assay system (Promega Corp., Madison, WI). Each of the indicated luciferase reporters, with the pRL-TK vector (Promega, Fitchburg, WI, USA) as an internal control, were transfected into human BC cells. After indicated concentration of ISO treatment for 24 h, cells were extracted with passive lysis buffer [25 mM Tris-phosphate (pH 7.8), 2 mM EDTA, 1% Triton X-100 and 10% glycerol]. The luciferase activity was measured using a microplate luminometer LB 96 V (Berthold GmbH & Co. KG, Bad Wildbad, Germany). The firefly luciferase signal was normalized to the Renilla luciferase transfection control.

### Statistical methods

Associations between categorical variables were assessed using the c2 test. The Student t-test was utilized to compare continuous variables, summarized as mean ± SD, between different groups. The paired *t*-test was performed to compare the difference between paired tissues using real-time PCR analysis. *P* < *0.05* was considered as significant difference.

## Results

### ISO treatment promoted RAC1 expression to induce autophagy and invasive inhibition in human invasive BC cells

**Fig. 1:**
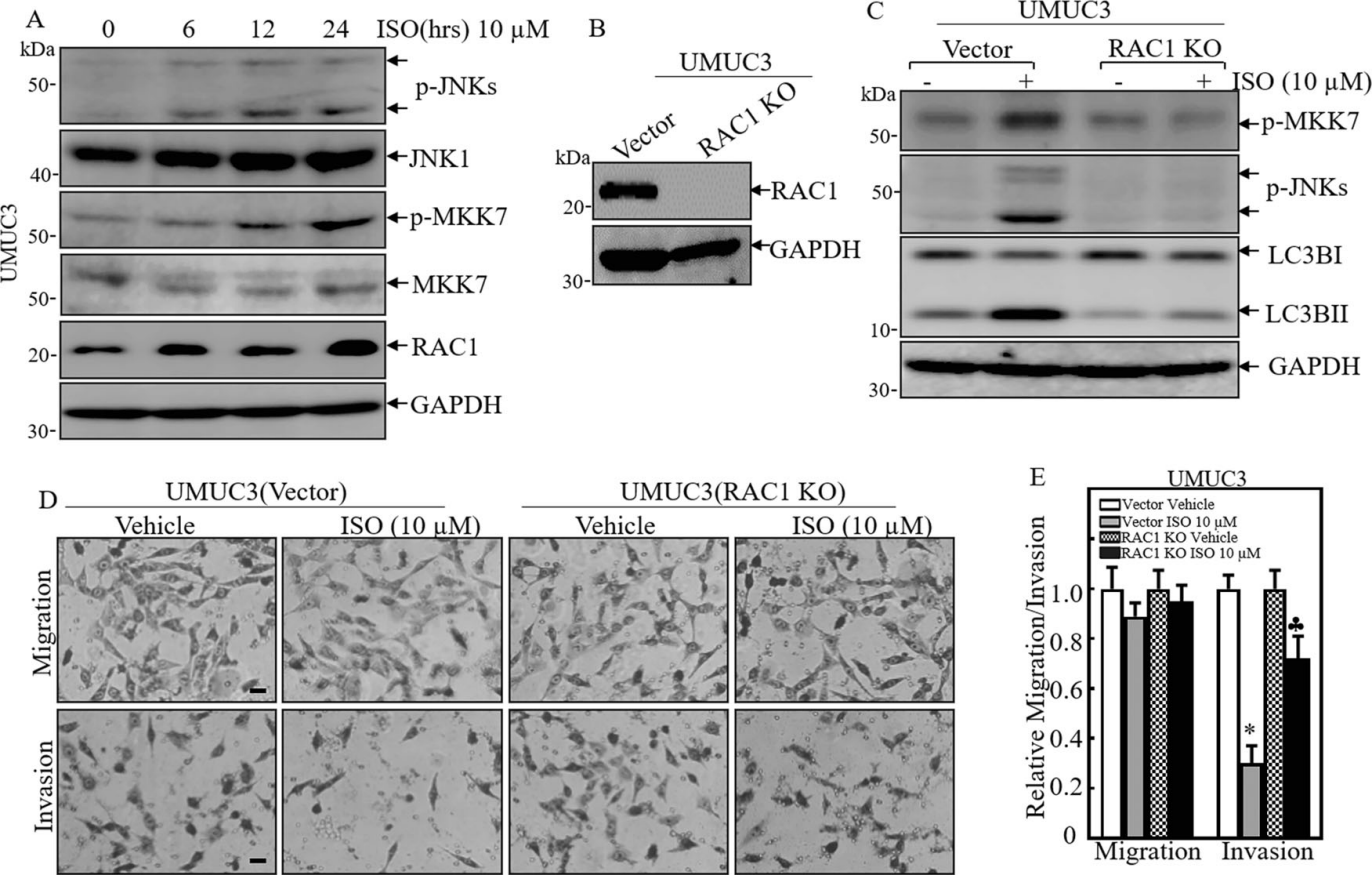
ISO treatment promoted RAC1 expression to induce autophagy and invasion inhibition in human invasive BC cells. **A** UMUC3 cells were treated with 10 μM of ISO for indicated time periods. The cells were then extracted and cell lysates were subjected to Western blot using the indicated antibodies. **B** CRISPR Cas9 for RAC1 was stably transfected into UMUC3 cells, and the stable clones were identified with Western blot. **C** The indicated stable cells were subjected to 10 μM of ISO treatment for 24 h for determination of the indicated proteins in UMUC3 cells. **D** UMUC3(RAC1 KO) and UMUC3(Vector) cells were subjected to transwell assay in medium containing either vehicle or 10 μM of ISO for 24 h. Representative images were captured under a microscope. **E** The invasion and migration rates were quantified by counting the relative migrated (Transwell) and invaded cells at least three random fields under a light microscope, and the bars show Mean ± SD from 3 independent experiments. The scale bar was 200 μM. ^*^Significant decrease from vehicle control (*p* < 0.05), ^♣^Significant increase from UNUC3(Vector) treated with ISO (*p* < 0.05).

Our recent study demonstrates that phosphorylated JNK is an upstream kinase responsible for ISO-induced JUN activation and SESN2-dependent autophagy as well as inhibition of anchorage-independent growth of human invasive BC cells [9]. To elucidate the upstream regulators responsible for ISO-induced JNK activation, we observed the potential JNK upstream regulators in ISO-treated UMUC3 cells. As shown in Fig. 1A, ISO treatment increased the phosphorylation of JNK and its upstream kinase MKK7 phosphorylation and RAC1 protein expression. RAC1 regulates a diverse array of cellular events, including the control of cell growth, cytoskeletal reorganization and the activation of protein kinases, such as JNKs [34]. To test whether RAC1 was responsible for ISO-mediated JNK phosphorylation and autophagic LC3B conversion, CRISPR/Cas9 specific targeting RAC1 were used to knockout *RAC1* gene in UMUC3 cells. The stable *RAC1* knockout transfectants were established and identified as shown in Fig. 1B. Knockout of *RAC1* abolished phosphorylation of JNK and its upstream MKK7 and autophagic LC3B conversion following ISO treatment in UMUC3 cells (Fig. 1C). Unexpectedly, our results also indicated that *RAC1* knockout attenuated the ISO inhibition of the invasion of UMUC3 cells as compared with that in the scramble vector transfectant UMUC3(Vector) cells (Fig. 1D, E). These results demonstrate that RAC1 induction contributes to both ISO-induced autophagic responses and the inhibition of invasion in human UMUC3 cells.

### SESN2-dependent autophagy mediated inhibition of human BC cell invasion

**Fig. 2:**
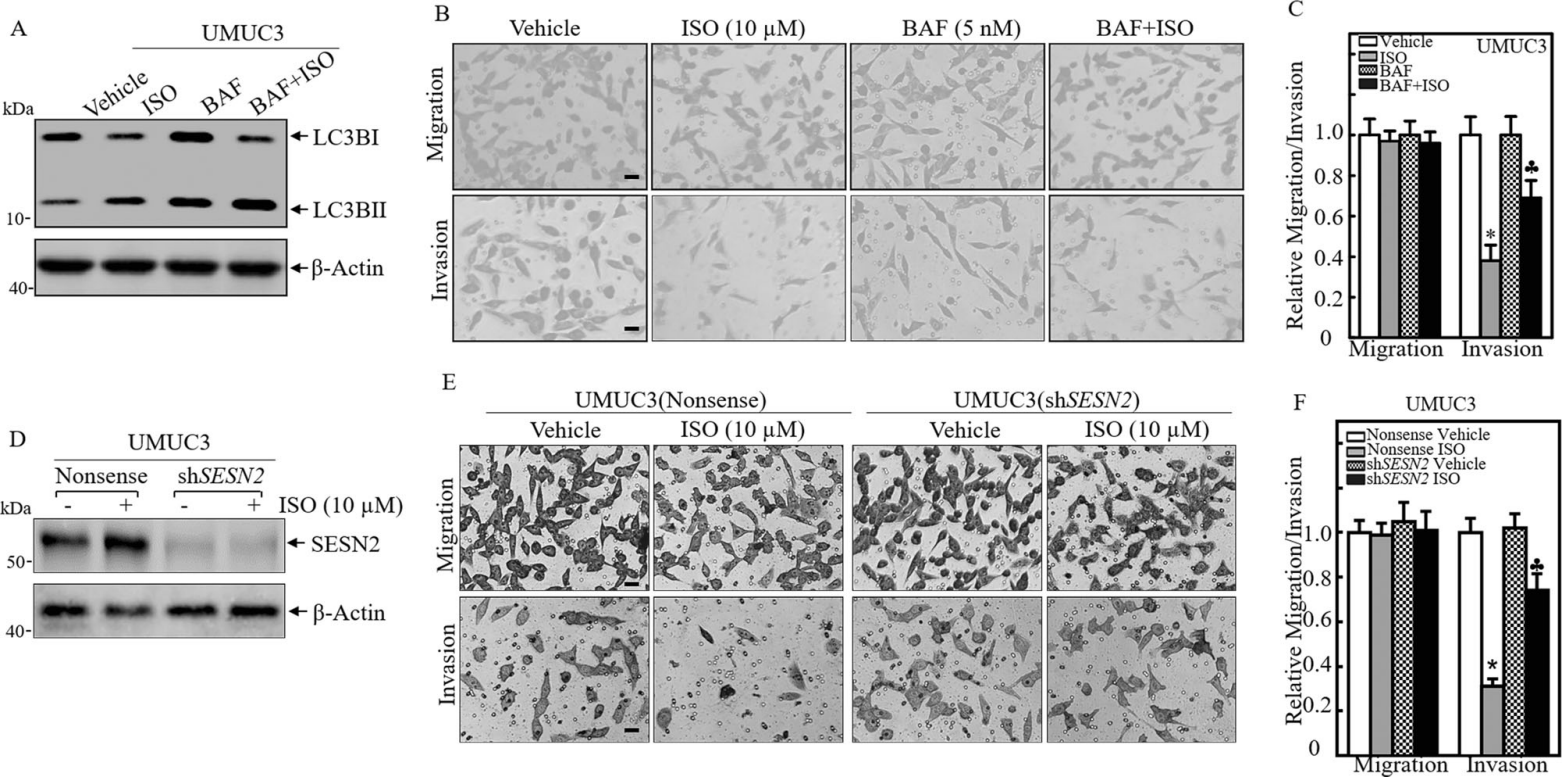
SESN2-dependent autophagy-mediated ISO inhibition of human BC cell invasion. **A** UMUC3 cells were treated with ISO (10 μM), with or without BAF (5 nM) for 24 h, and the cell extracts were subjected to Western blot for determining of conversion of LC3B I to LC3B II. **B**, **C** Representative images of migration and invasion of UMUC3 treated with 10 μM ISO, 5 nM BAF or both in cellular migration and invasion assay were captured under a microscope (**B**). The invasion and migration cells were counted at least three random fields under a light microscope and the migration (Transwell) and invasion rates were calculated as relative to vehicle control as described in the “Materials and Methods” section. The bars indicate Mean ± SD from three independent experiments. ^*^Significant decrease from vehicle control (*p* < 0.05), ^♣^Significant increase from ISO-treated alone (*p* < 0.05). **D** The indicated stable transfectants were subjected to Western blot for determination of SESN2 expression with or without 10 μM of ISO treatment for 24 h in UMUC3 cells. **E**, **F** The stable transfectants, UMUC3(sh*SESN2*), were used for determination of their abilities of cell invasion and migration as compared with their vector control transfectants as described above. The bars indicate Mean±SD from 3 independent experiments. ^*^Significant decrease from UMUC3(Nonsense) treated with vehicle control (*p* < 0.05). ^♣^Significant increase from UMUC3^(^Nonsense) treated with ISO alone (*p* < 0.05).

Above results indicated that RAC1 contributed to both autophagic induction and invasion inhibition by ISO, suggesting there might be association of autophagic induction and invasion inhibition. To test this possibility, we first evaluated the effects of autophagy chemical inhibitor Bafilomycin A1 (BAF) on the invasive inhibition. The results showed that ISO treatment led to a remarkable inhibition of BC invasion without affecting cell migration, whereas inhibition of ISO-induced autophagy by BAF (Fig. 2A) abolished ISO inhibition of UMUC3 cell invasion (Fig. 2B, C). These results suggest that the autophagic induction might be associated with ISO inhibition of BC invasion. To approve this notion, the potential effects of SESN2 depletion on ISO-mediated inhibition of invasion were evaluated, and the results showed that *SESN2* knockdown pronouncedly reversed the inhibitory effects of ISO on the invasion of human BC cells (Fig. 2D–F). Collectively, our results reveal that SESN2 plays an important role in ISO-mediated autophagy induction and BC invasive inhibition.

### Downregulation of miR-365a by ISO contributed to an increased RAC1 protein translation, JNK phosphorylation and invasion inhibition in human invasive BC cells

**Fig. 3:**
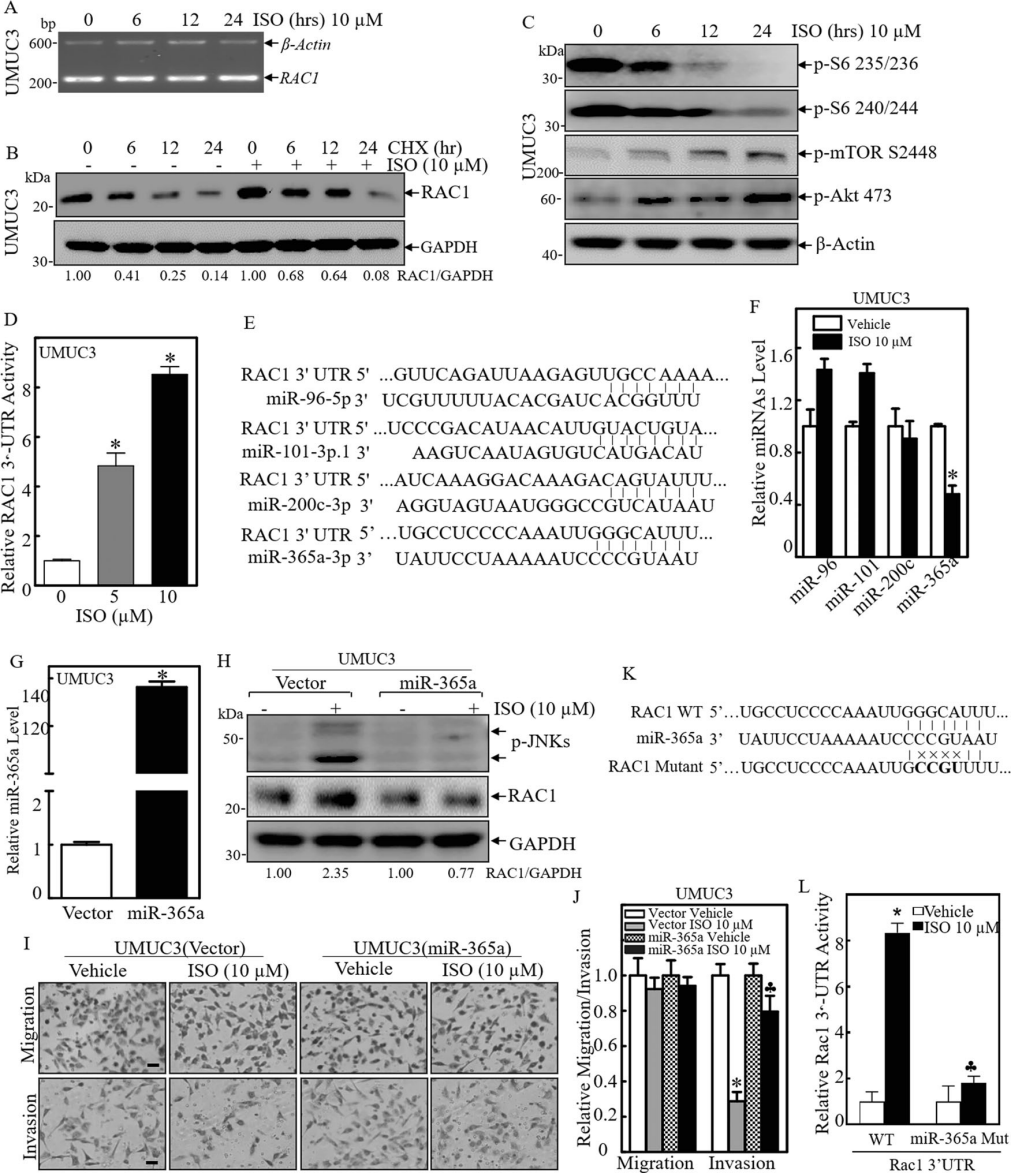
Downregulation of miR-365a contributed to increased RAC1 protein translation, JNK phosphorylation and invasion inhibition following ISO treatment. **A** Total RNAs were isolated from UMUC3 cells treated with or without 10 μM of ISO for indicated time periods. RT-PCR was performed to determine *RAC1* mRNA levels. The *β-Actin* mRNA levels were used as a loading control. **B** UMUC3 cells were treated with cycloheximide (CHX; 100 µg/ml) combined with ISO (10 μM) or CHX alone as indicated time periods. The cell extracts were then subjected to Western blot for determining RAC1 expression and GAPDH protein expression was used as a protein loading control. **C** UMUC3 cells were treated with 10 μM of ISO for the indicated time periods. The cell extracts were subjected to Western blot to evaluate S6 phosphorylation at Ser235/236 and Ser240/244, mTOR phosphorylation at Ser2448, AKT phosphorylation at Ser473. β-Actin was used as a protein loading control. **D** RAC1 3′-UTR luciferase reporter was transiently transfected into UMUC3 cells, and the transfectants were subjected to determine the effect of ISO on *RAC1* mRNA 3′-UTR activity in UMUC3 cells following 5 and 10 μM of ISO treatment for 24 h. The results were shown as Mean±SD from three independent experiments. ^*^Significant increase of RAC1 3′-UTR activity in comparison to vehicle control (*p* < 0.05). **E** The potential binding sites of miRNAs in 3′-UTR of human *RAC1* mRNA were analyzed and presented as indicated. **F** The relative expression levels of miRNAs were evaluated by quantitative real-time PCR in UMUC3 cells followed by ISO (10 μM) treatment for 24 h. **G** Over-expression of miR-365a in UMUC3 cells was evaluated by real-time PCR assay. **H** The indicated stable transfectants were subjected to Western blot for determination of JNK activation and RAC1 expression with or without ISO (10 μM) treatment for 24 h in UMUC3 cells. (**I**, **J**) UMUC3(miR-365a) and UMUC3(Vector) were then used for determination of their migration and invasion abilities in the presence of either vehicle or 10 μM of ISO treatment for 24 h. **K** Schematic of the construction of miR-365a binding site mutant of the pMIR-Report-*RAC1* mRNA 3’-UTR luciferase reporter. ^*^Significant decrease from UMUC3(Vector) treated with vehicle control (*p* < *0.05*). ^♣^Significant increase from UMUC3(miR-365a) cells (*p* < *0.05*). **L** Attenuation of miR-365a inhibition of *RAC1* mRNA 3′-UTR luciferase reporter activity in miR-365a binding site mutant of pMIR-Report-*RAC1* 3’-UTR transfectants. Results were presented as the Mean±SD of triplicates. ^*^Significant increase from WT-reporter transfectants treated with vehicle control (*p* < 0.05), ^♣^Significant decrease from WT-reporter transfectants treated with ISO alone (*p* < 0.05).

The upregulation of RAC1 protein expression by ISO treatment could occur at levels of transcription, mRNA stability, protein translation or protein degradation. We, therefore, examined the potential effects of ISO on *RAC1* mRNA expression. The results indicated that ISO treatment did not have any observable effects on *RAC1* mRNA expression level (Fig. 3A), thereby excluding the possibility that ISO treatment modulates RAC1 expression at regulation of transcriptional or mRNA stability levels. To test whether ISO treatment affects RAC1 expression at the post-transcriptional level, we determined the effects of ISO on RAC1 protein degradation rate. Unexpectedly, ISO treatment slightly accelerated RAC1 protein degradation as shown in Fig. 3B, further suggesting that ISO treatment might regulate RAC1 protein translation. Given that ribosome biogenesis is a fundamental process that provides cells with the molecular factories for cellular protein translation [35] and phosphorylation of the 40 S ribosomal protein S6 at Ser235/236 plays an important role in the regulation of protein translation [36], we evaluated the phosphorylation of S6 at Ser235/236 in UMUC3 cells following ISO treatment. The results from Western blot showed that ISO treatment attenuated S6 phosphorylation at Ser235/236 (Fig. 3C), excluding the possibility that ISO upregulates RAC1 protein translation through ribosomal protein S6 canonical pathway.

microRNAs (miRNAs) have been reported to inhibit their target gene expression by suppressing protein translation usually through imperfect complementary base pairing to the target gene mRNA 3′UTR [37]. We, therefore, tested the potential effect of ISO on *RAC1* mRNA 3′-UTR activity. It was noted that ISO treatments at a dose of 5 and 10 µmol/L could markedly induce *RAC1* mRNA 3′-UTR activity in UMUC3 cells (Fig. 3D). With that result in mind, we further used the TargetScan and miRCode database to analyze potential miRNA binding sites in the 3′-UTR regions of *RAC1* mRNA. As shown in Fig. 3E, miR-96, miR-101, miR-200c, and miR-365a, could have potential binding to 3′-UTR of *RAC1* mRNA. To identify which of these miRNAs was involved in the regulation of RAC1 protein translation, we evaluated these miRNAs’ expression in UMUC3 treated with ISO. The results showed that ISO treatment decreased miR-365a expression, slightly increased miR-6 and miR-101, and had no effect on miR-200c in UMUC3 cells (Fig. 3F), suggesting that miR-365a might be involved in ISO promoting RAC1 protein translation in UMUC3 cells.

To test the role of miR-365a in the regulation of ISO-induced RAC1 protein translation, miR-365a was stably transfected into UMUC3 cells, and the ectopic expression level of miR-365a was evaluated by real-time PCR as shown in Fig. 3G. Ectopic overexpression of miR-365a resulted in a marked inhibition of JNK phosphorylation and RAC1 expression, as well as reversed ISO inhibition of invasion in UMUC3 cells (Fig. 3H–J). To define whether miR-365a inhibition of *RAC1* mRNA 3’-UTR activity was due to its specific binding to its binding site at 3’-UTR region of *RAC1* mRNA, we constructed mutant of RAC1 3’-UTR luciferase reporter as displayed in Fig. 3K. Both WT and mutant of *RAC1* mRNA 3′-UTR luciferase reporters were stably transfected into UMUC3 cells, and the transfectants were employed to evaluate the effects of ISO on the reporter activity. As shown in Fig. 3L, ISO treatment profoundly promoted WT *RAC1* mRNA 3′-UTR activity, whereas mutation of miR-365a binding site impaired the effect of ISO on *RAC1* mRNA 3′-UTR activity, demonstrating that the miR-365a binding site in *RAC1* mRNA 3′-UTR is critical for ISO induction of *RAC1* mRNA 3′-UTR activity. Taken together, our results indicate that ISO treatment inhibits miR-365a expression, which results in the reduction of miR-365a binding to 3′-UTR of *RAC1* mRNA and subsequently increasing RAC1 protein translation, thereby activating MKK7/JNK-dependent autophagy and leading to the inhibition of human BC cell invasion.

### SOX2 inhibition mediated the reduction of miR-365a transcription and expression

**Fig. 4:**
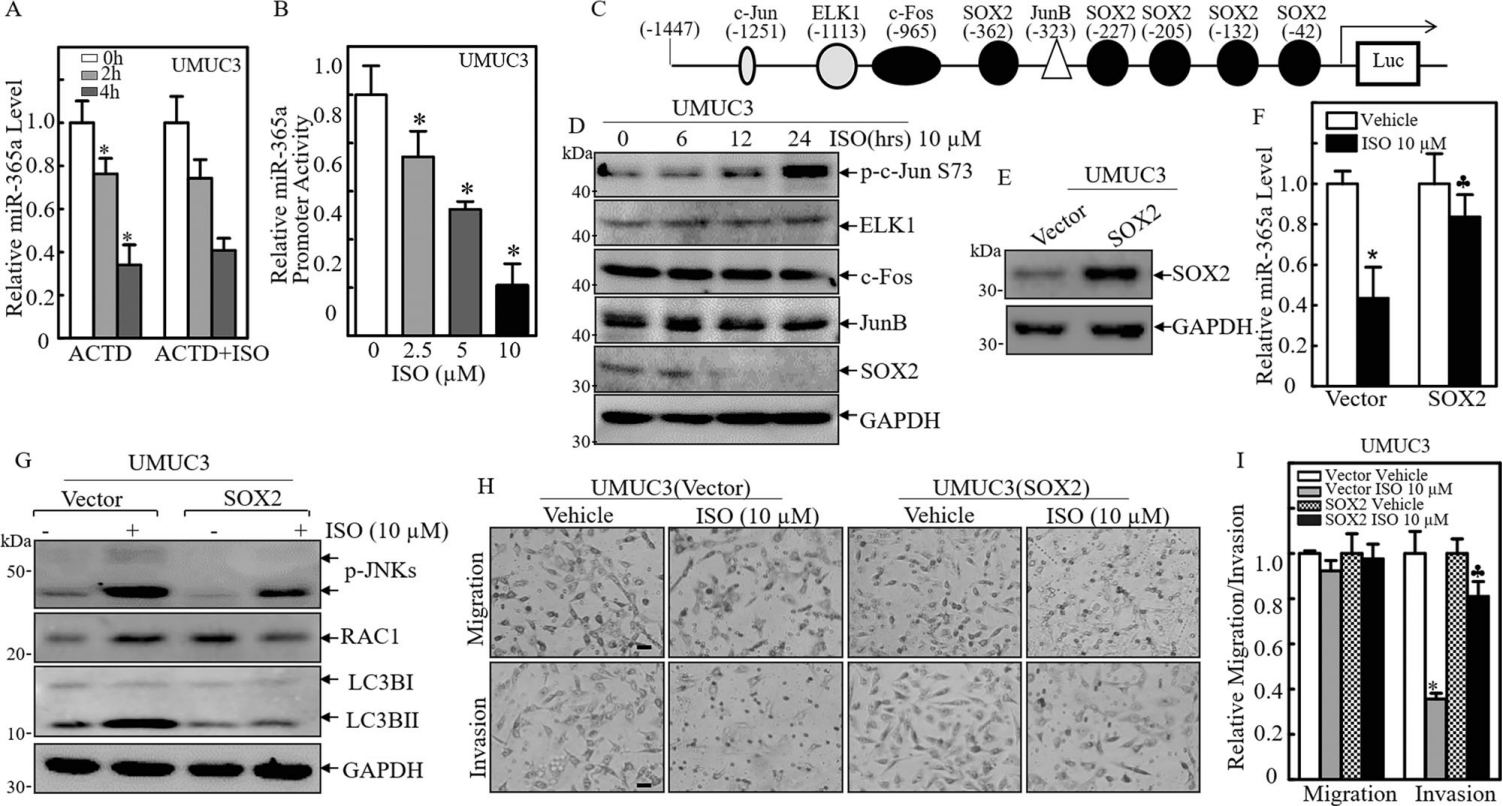
ISO treatment attenuated SOX2 expression and, in turn, reduced miR-365a transcription and expression. **A** UMUC3 cells were co-incubated with ISO (10 μM) and Act D (20 μg/ml) for indicated time periods. Total RNA was isolated and quantitative real-time PCR was then performed to determine miR-365a levels. *GAPDH* was used as an internal control. Results were presented as the Mean±SD of triplicates. ^*^Significant decrease from vehicle control (*p* < 0.05). **B** UMUC3 stably transfected with miR-365a promoter-driven luciferase reporter was treated with ISO at different concentrations for 24 h to determine the potential effect of ISO on miR-365a promoter transcriptional activity. **C** Schematic representation of the putative transcription factor consensus binding sites in the miR-365a proximal promoter region **D** The protein expressions of potential transcription factors was determined by Western blot in UMUC3 cells treated with ISO (10 μM) for indicated time periods. **E** Over-expression of SOX2 constructs were stably transfected into UMUC3 cells and the stable transfectants were identified by Western blot. **F** Over-expression of SOX2 reversed the inhibition effect of ISO on miR-365a relative expression in UMUC3 cells. **G** Over-expression of SOX2 reversed ISO-induced RAC1, p-JNK and LC3B-II protein abundance in UMUC3 cells. **H**, **I** UMUC3(SOX2) and UMUC3(Vector) were used for determination of their migration and invasion abilities in the presence of either vehicle or 10 μM of ISO treatment for 24 h. Results were presented as the mean ± SD of triplicates. ^*^Significant difference between vector control and overexpression group (*p* < 0.05). ^♣^Significant increase from UMUC3(Vector) treated with ISO alone (*p* < 0.05).

To characterize the molecular mechanism underlying miR-365a inhibition by ISO treatment, the potential effect of ISO on miR-365a stability was determined. UMUC3 cells were treated with actinomycin D (Act D, 20 μg/ml) with or without ISO for indicated time periods, and real-time PCR was employed to evaluate miR-365a abundance. The result showed that in comparison to miR-365a level observed in UMUC3 cells treated with Act D alone, the cells treated with combination of Act D and ISO did not show observable change of miR-365a degradation rate (Fig. 4A), excluding the possibility of ISO affecting miR-365a stability. Thus, the miR-365a promoter-driven luciferase reporter was constructed and transfected into UMUC3 cells. Luciferase reporter assay was performed to determine the miR-365a promoter activity in the cells treated with ISO. As seen in Fig. 4B, ISO treatment led to a dramatic inhibition of miR-365a promoter transcription activity in a dose-dependent manner. To identify the transcription factor(s) responsible for ISO downregulation of miR-365a transcription, PROMO website was employed to bioinformatically analyze the miR-365a promoter region. The results showed that there were several putative transcription factor DNA-binding sites, including AP-1 (c-Jun, c-Fos, JunB), ELK1, and SOX2 in the miR-365a promoter region, as shown in Fig. 4C. To identify the specific transcription factor(s) participating in the modulation of miR-365a transcription, the effect of ISO on the related transcription factor protein abundance/activation was determined. The results revealed that ISO treatment clearly inhibited SOX2 expression, induced c-Jun activation and had no effect on ELK1, c-Fos, and Jun B (Fig. 4D). To evaluate whether SOX2 was involved in ISO-inhibited miR-365a transcription, the constructs that exogenous overexpress SOX2 and its corresponding vector were stably transfected into UMUC3 cells, and the stable transfectants were identified as shown in Fig. 4E. Overexpression of SOX2 attenuated ISO inhibition of miR-365a expression as compared with that in UMUC3(Vector) (Fig. 4F). Consistently, SOX2 overexpression resulted in a remarkable inhibition of JNK phosphorylation, RAC1 expression, autophagy induction, and reversed the inhibition of invasive ability of UMUC3 cells followed ISO treatment (Fig. 4G–I).

### miR-145 induction by ISO inhibited SOX2 expression and invasion in human BC cells

**Fig. 5:**
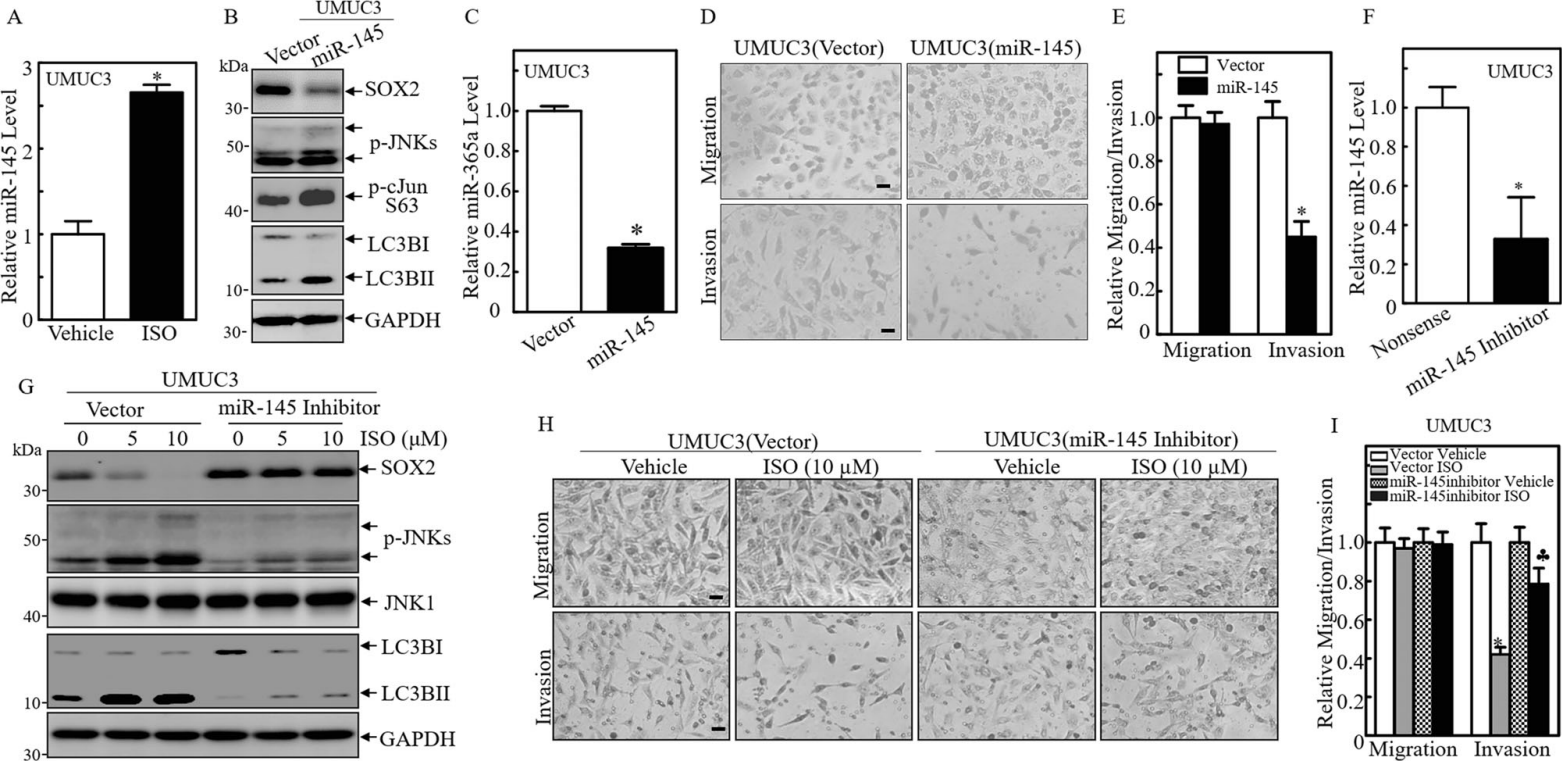
miR-145 induction was crucial for ISO inhibition of SOX2 expression and invasion in BC cells. **A** The relative expression level of miR-145 was evaluated by quantitative real-time PCR in UMUC3 cells followed by 10 µM ISO treatment for 24 h. **B** The cell extracts from UMUC3(vector) and UMUC3(miR-145) transfectants were subjected to Western blot for determination of protein expressions as indicated. **C** The relative expression level of miR-365a was evaluated by quantitative real-time PCR in UMUC3(miR-145) and its vector control cells. **D**, **E** UMUC3(miR-145) and Nonsense transfectant UMUC3(Vector) were then used for determination of their migration and invasion abilities. Results are the Mean±SD of triplicates. *Significant difference between vector control and overexpression group (*p* < 0.05). **F** UMUC3 cells were stably transfected with miR-145 inhibitor or its control vector, and the relative miR-145 expression level was determined by quantitative real-time PCR. ^*^Significant difference (*p* < 0.05). **G** UMUC3 cell transfectants as indicated were treated with 5 and 10 μM of ISO for 24 h, and the cell extracts were subjected to Western blot for determination of protein expressions. **H**, **I** UMUC3(miR-145 inhibitor) and UMUC3(Vector) were then used for determination of their migration and invasion abilities in the presence of either vehicle or 10 μM of ISO treatment for 24 h. Results are the Mean ± SD of triplicates. ^*^Significant difference between vector control and overexpression group (*p* < 0.05). ^♣^Significant increase from UMUC3(Vector) treated with ISO alone (*p* < 0.05).

Our previous studies show that ISO treatment induces miR-145 expression in both human BC cells and human glioblastoma cells [18, 38], Moreover, in human glioblastoma cells, ISO treatment inhibits SOX2 mRNA 3’UTR activity; and the point mutation of miR-145 binding site in SOX2 mRNA 3’UTR also reveals that miR-145 inhibits SOX2 protein translation by binding directly to the SOX2-3’UTR region [38]. To test whether miR-145 induction by ISO was responsible for inhibition of SOX2 expression in human BC cells, qPCR was employed to evaluate the effect of ISO on miR-145 expression in UMUC3 cells. The results indicated that ISO treatment led to a significant miR-145 induction (Fig. 5A). To test whether miR-145 itself could mimic the biological effects of ISO that were observed in UMUC3 cells, miR-145 expressing construct was transfected into UMUC3 cells. Over-expression of miR-145 impaired expression of SOX2 and miR-365a, increased the activation of JNK and c-Jun, as well as induction of autophagy (Fig. 5B, C). Over-expression of miR-145 also inhibited invasive ability in UMUC3 cells (Fig. 5D, E). Consequently, knockdown of miR-145 expression using its specific inhibitor increased SOX2 expression, abolished JNK activation and autophagy induction, as well as reversed ISO inhibition of BC cell invasion in UMUC3 cells (Fig. 5F–I). These results demonstrate that miR-145 induction by ISO mediates its inhibition of SOX2 expression and miR-365a transcription, further inhibiting BC cell invasion.

### ISO treatment induced Dicer protein translation and promoted miR-145 maturation and expression in human BC cells

**Fig. 6:**
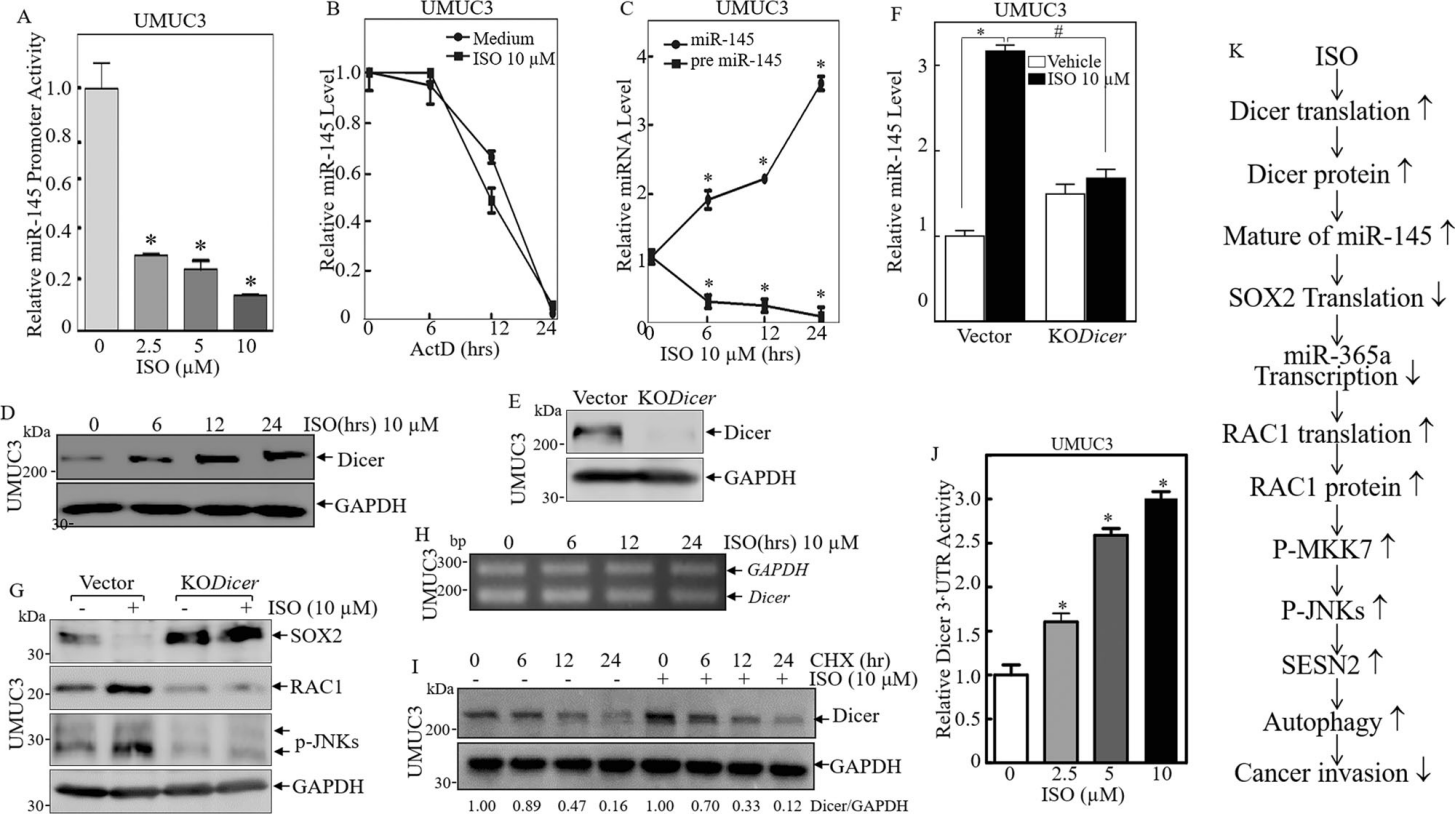
ISO treatment accumulated Dicer and in turn, upregulated miR-145 maturation and expression. **A** UMUC3 stably transfected with miR-145 promoter-driven luciferase reporter was treated with ISO at different concentrations for 24 h to determine the potential effect of ISO on miR-145 promoter transcriptional activity. **B** UMUC3 cells were co-incubated with ISO (10 μM) and Act D (20 μg/ml) for indicated time periods. Total RNA was isolated and quantitative real-time PCR was then performed to determine miR-145 levels. *GAPDH* was used as internal control. The result was a representative one from three independent experiments. **C** The relative expression levels of pre-mir-145 and miR-145 were evaluated by quantitative real-time PCR in UMUC3 followed by ISO treatment at different concentrations for 24 h. ^*^Significant change from vehicle control (*p* < 0.05). **D** UMUC3 cells were treated with various concentrations of ISO for 24 h. The cell lysates were subjected to Western blot to detect Dicer protein expression. **E** CRISPR/Cas9 for Dicer was stably transfected into UMUC3 cells, and the stable cells were identified with Western blot. **F** UMUC3(KO*Dicer*) and UMUC3(Vector) cells were treated with or without 10 μM of ISO for determination of miR-145 level using Real-time PCR. **G** Cell extracts from UMUC3(KO*Dicer*) and UMUC3(Vector) were subjected to Western blot to evaluate the downstream protein expression following treatment with 10 μM of ISO for 24 h. **H** Total RNA was isolated from the UMUC3 cells treated with 10 μM of ISO for indicated time periods. RT-PCR was performed to determine *Dicer* mRNA levels. The *GAPDH* mRNA levels were used as loading control. **I** UMUC3 were pre-treated with MG132(10 μM) for 6 h, and the cells were then incubated with CHX (100 µg/ml) for the indicated time periods with or without ISO (10 μM). The cell extracts were then subjected to Western blot to analyze Dicer protein degradation rates. GAPDH was used as a protein loading control. **J** Dicer 3′-UTR luciferase reporter was transiently transfected into UMUC3 cells, and the transfectants were subjected to determine the effect of ISO on *Dicer* mRNA 3′-UTR activity in UMUC3 cells following ISO treatment for 24 h. ^*^Significant increase of Dicer 3′-UTR activity in comparison to vehicle control (*p* < 0.05). The results were shown as Mean ±  SD from three independent experiments. **K** The schematic summary of molecular mechanisms underlying the anti-cancer activity of ISO on human bladder cancer invasion.

To elucidate the molecular mechanism leading to miR-145 induction by ISO treatment, the miR-145 promoter-driven luciferase reporter was constructed and transfected into UMUC3 cells. As shown in Fig. 6A, ISO treatment dramatically inhibited miR-145 promoter transcription activity, which was inconsistent with ISO promotion of miR-145 expression, further excluding the possibility of ISO activating miR-145 transcription. It is reported that miRNAs expression could be regulated at stability in the intact cells [39]. Thus, the potential effect of ISO on miR-145 stability was, therefore, determined. UMUC3 cells were treated with Act D (20 μg/ml) alone or together with ISO for indicated time periods, and real-time PCR was performed to determine miR-145 abundance. The result showed that the cells treated with combination of Act D and ISO did not show observable changes of the miR-145 degradation rate in comparison to that observed in cells treated with Act D alone (Fig. 6B). These results led us to explore the possibility of ISO modulation of pre-mir-145 maturation with considering miRNA biogenesis process. Therefore, we compared levels of pre-mir-145 and mature miR-145 expression in UMUC3 cells treated with ISO. As expected, pre-mir-145 was remarkably reduced, accompanied with induction of mature miR-145 in ISO-treated UMUC3 cells in a time-dependent manner (Fig. 6C), revealing that ISO treatment exhibited an accelerative effect on pre-mir-145 maturation. Some pre-miRNA maturation needs successive enzymatic Dicer to cleave pre-miRNA into mature miRNA [40]. We hence tested whether ISO treatment affected Dicer expression, by which increase in pre-mir-145 maturation. The results showed that Dicer protein was significantly increased in the UMUC3 cells treated with ISO (Fig. 6D). To test whether elevated Dicer by ISO contributed to promoting miR-145 maturation, we knocked out Dicer in UMUC3 cells using CRISPR/Cas9 (Fig. 6E). *Dicer* knockout impaired miR-145 maturation following ISO treatment (Fig. 6F). *Dicer* knockout also reversed ISO inhibition of SOX2 expression, as well as induction of RAC1 expression and JNK phosphorylation (Fig. 6G). These results suggest that Dicer is critical for miR-145 maturation following ISO treatment in human BC cells.

To explore how ISO regulates Dicer expression, we tested *Dicer* mRNA level in ISO-treated UMUC3 cells. As shown in Fig. 6H, ISO treatment didn’t affect its mRNA levels in the UMUC3 cells, suggesting that ISO-mediated Dicer abundance may occur at the post-transcriptional level. Following this, the Dicer protein degradation rate was then evaluated. As seen in Fig. 6I, the cells treated with cycloheximide (CHX) alone or together with ISO showed a similar Dicer protein degradation rates, excluding the possibility that ISO regulates Dicer protein degradation. Collectively with Fig. 6J, these results demonstrate that acceleration of pre-mir-145 maturation by ISO is through promoting Dicer protein translation in human BC cells.

## Discussion

Muscle invasive bladder cancer (MIBC) accounts for virtually all the mortality from BC and represents a major therapeutic challenge of this disease [41]. Therefore, an endeavor to identify new anticancer compounds with strong inhibition of cancer invasion and understand the mechanisms underlying their inhibitory effect on BC invasion are the key step to discover the unmet future medicines against the invasive malignant BCs. The naturally occurring compound ISO has been proved to possess anti-cancer activities against multiple human cancer cells [5, 42], such as BCs [9], glioblastoma [38] and prostate cancer [43]. Our results obtained from in vivo animal studies show that ISO treatment inhibits over 90% of BBN-induced mouse MIBC formation, for the first time demonstrating the chemo-preventive effects of ISO on MIBCs to the best of our knowledge [8]. Here, we found that ISO at in vivo–relevant concentrations of 10 µmol/L represses BC invasion by the induction of autophagy through promoting RAC1/JNK pathway. Our study also identified that RAC1 induction by ISO is due to transcriptional downregulation of miR-365a, which can directly bind to *RAC1* mRNA 3’-UTR region and inhibits RAC1 protein translation. Further, transcriptional downregulation of miR-365a is mediated by translational inhibition of SOX2 by ISO treatment, whereas translational inhibition of SOX2 is caused by induction of miR-145 through its direct binding to *SOX2* mRNA 3’UTR region, which is demonstrated in our recent study [38]. Moreover, we showed that ISO-initiated Dicer protein expression exhibits a positive effect on pre-mir-145 maturation and induction, as well as SOX2/miR365a/RAC1/MKK7/JNK-dependent autophagy and BC invasion inhibition. Collectively, our results reveal that the natural compound ISO induces autophagy-dependent inhibition of human BC invasion through targeting Dicer/miR-145/SOX2/miR-365a/RAC1/MKK7/JNK/SESN2 axis as diagramed in Fig. 6K.

The endoribonuclease III Dicer is known for its critical role in the canonical biogenesis of eukaryotic small RNAs, especially microRNAs in multiple RNA interference pathways. However, more and more evidence show that some canonical miRNAs were still produced without Dicer albeit at markedly reduced levels [44, 45]. In other words, the Dicer-dependent pathway is not necessary for all miRNA maturation, which has also been verified in our recent publication [46]. It has been reported that Dicer exhibits aberrant expression in several cancer types and involves in many pathological cellular processes, such as cancer cell growth, invasion, EMT, and metastasis [46–49]. The pleiotropic role of Dicer in tumorigenesis is not only dose-dependent but also tissue context-dependent. In lung [50], breast [51], and ovarian cancers [52], lower Dicer expression was found to be associated with advanced tumor stages and poor clinical outcome. Conversely, Dicer is up-regulated in human prostate cancers and knocking down Dicer expression suppresses the growth and tumorigenic capacity but leads to an increase in apoptosis and senescence [47]. Moreover, the response of Dicer to some different anti-cancer drugs is also divergent. Giovanni et.al. demonstrate that metformin induces Dicer expression, which plays an important role in the anticancer metabolic effects of metformin in breast cancer [53]. It is also reported that low Dicer levels are associated with better response to Bevacizumab-based treatments in advanced colorectal cancer patients [54]. Our most recent studies reveal that ISO treatment upregulates Dicer protein expression in muscle-invasive human bladder cancer T24T cells, which further promoting miR-4295 maturation and ultimately suppress the stem-like properties of the human BC cells [55]. Here, we found that ISO also enhances Dicer protein expression in human bladder cancer UMUC3 cells. The increased Dicer promotes miR-145 maturation and subsequent inhibits autophagic cell invasion.

Due to the context-dependent complexity of autophagy, it has been considered as a double-edged sword that either promote or suppress human cancers, depending on the stimuli of autophagy, the stage of cancer, and the downstream mediators or effectors, making anti-cancer therapeutic approaches highly challenging [56, 57]. Our recent studies have shown that autophagic responses mediated by Sestrin 2 (SESN2) following anti-cancer compounds ISO or ChlA-F treatment mainly results in autophagic inhibition of human bladder cancer cell growth [9, 58], whereas ATG7-mediated autophagic responses promote growth and invasion of human bladder cancer cells [59, 60]. Therefore, it is essential and of high significance to identify the functional types of autophagy induced by different initiators as well as the underlying mechanisms prior to the application in clinical trials. In current study, we associated SESN2-mediated autophagy induction with BC invasion inhibition by ISO and identified an upstream regulatory axis, Dicer/miR145/SOX2/miR365a/RAC1, leading to MKK7/JNKs activation and autophagy induction.

In conclusion, we have shown that ISO treatment suppressed human BC cell invasion accompanied by upregulation of RAC1 protein translation and its downstream target MKK7/JNK phosphorylation/activation, in turn leading to BC cell autophagy. We found that ISO treatment results in RAC1 protein up-regulation, and inhibited BC cell invasion, while knockout of *RAC1* profoundly reversed ISO inhibition of BC invasion. Further studies showed that ISO up-regulation of RAC1 translation was mediated by the inhibition of miR-365a, which can directly bind to the 3’UTR of *RAC1* mRNA. The attenuation of miR-365a was caused by inhibition of its upstream transcription factor SOX2 by ISO *via* promoting maturation and expression of miR-145, which has been reported to direct binding to *SOX2* mRNA 3’-UTR and inhibiting SOX2 protein translation. Finally, we found that Dicer protein translation was remarkably increased by ISO treatment, and in turn, promoting miR-145 maturation. These findings not only provide a novel insight linking autophagy induction to ISO inhibition of BC invasion, but also identify upstream Dicer/miR-145/SOX2/miR365a/RAC1 axis activating MKK7/JNK/SESN2 leading to autophagic responses in human BC cells following ISO treatment.

## Supplementary information

Original Data File

aj-checklist

author contribution

## Data Availability

All data generated or analysed during this study are included in this published article.
