# Supplementary material for: Induction of RAC1 protein translation and MKK7/JNK-dependent autophagy through dicer/miR-145/SOX2/miR-365a axis contributes to isorhapontigenin (ISO) inhibition of human bladder cancer invasion
Source: Cell Death Dis. 2022 Aug 31;13(8):753. doi: 10.1038/s41419-022-05205-w (PMC9433410; doi:10.1038/s41419-022-05205-w)
Supplement: Supplementary file 1 — Original Data File [file 41419_2022_5205_MOESM1_ESM.pdf]

Figure 1

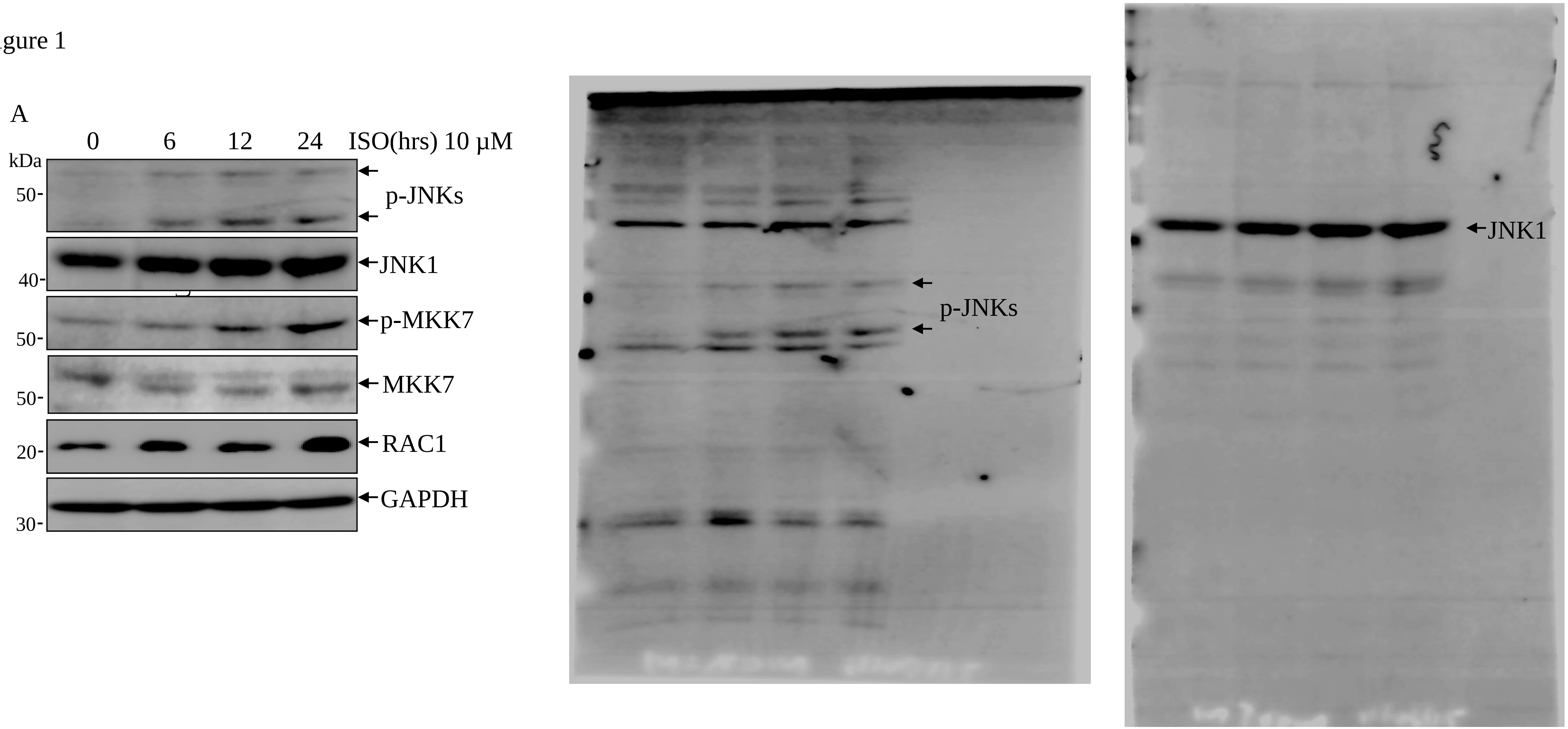
A
0
6
12
24
ISO(hrs) 10 μM
kDa
50-
p-JNKs
JNK1
40-
p-MKK7
50-
MKK7
50-
RAC1
20-
GAPDH
30-
p-JNKs
JNK1

Figure 1

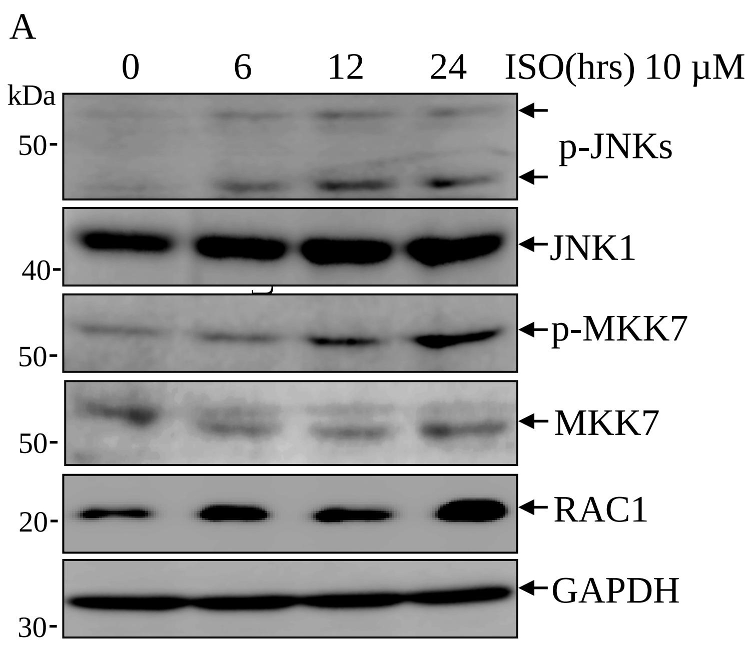
A
0
6
12
24
ISO(hrs) 10 µM
kDa
50-
40-
50-
50-
20-
30-
p-JNKs
JNK1
p-MKK7
MKK7
RAC1
GAPDH

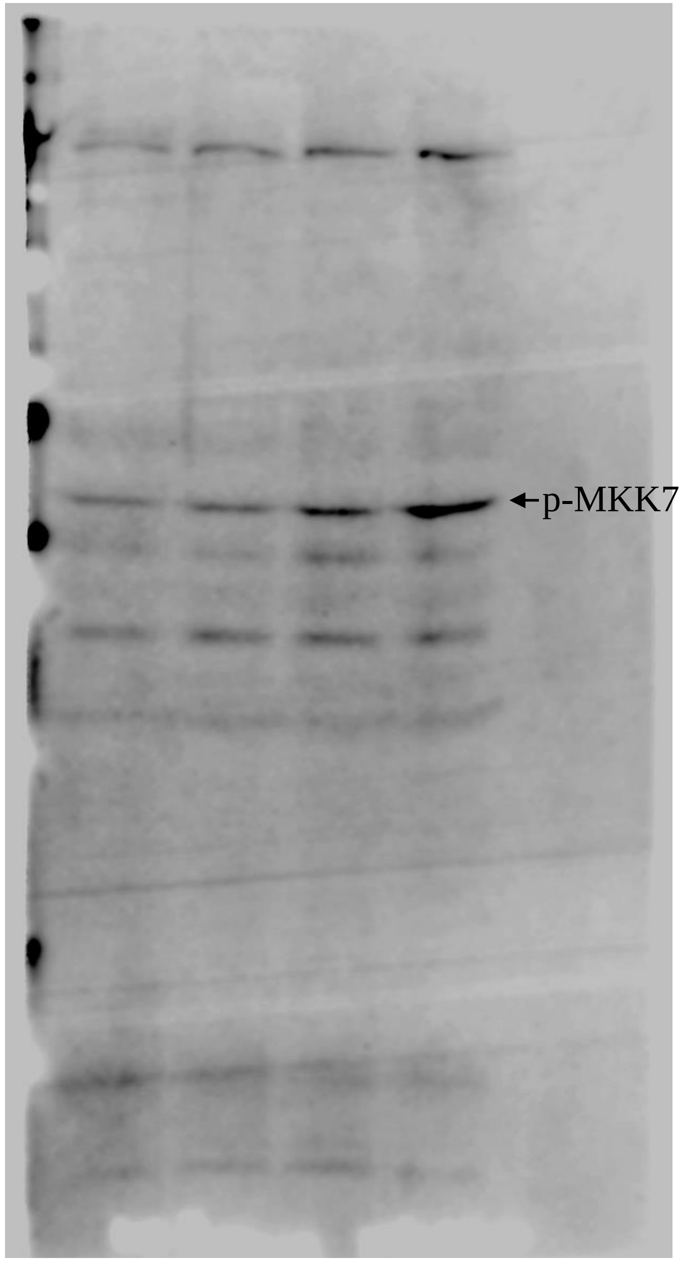
p-MKK7

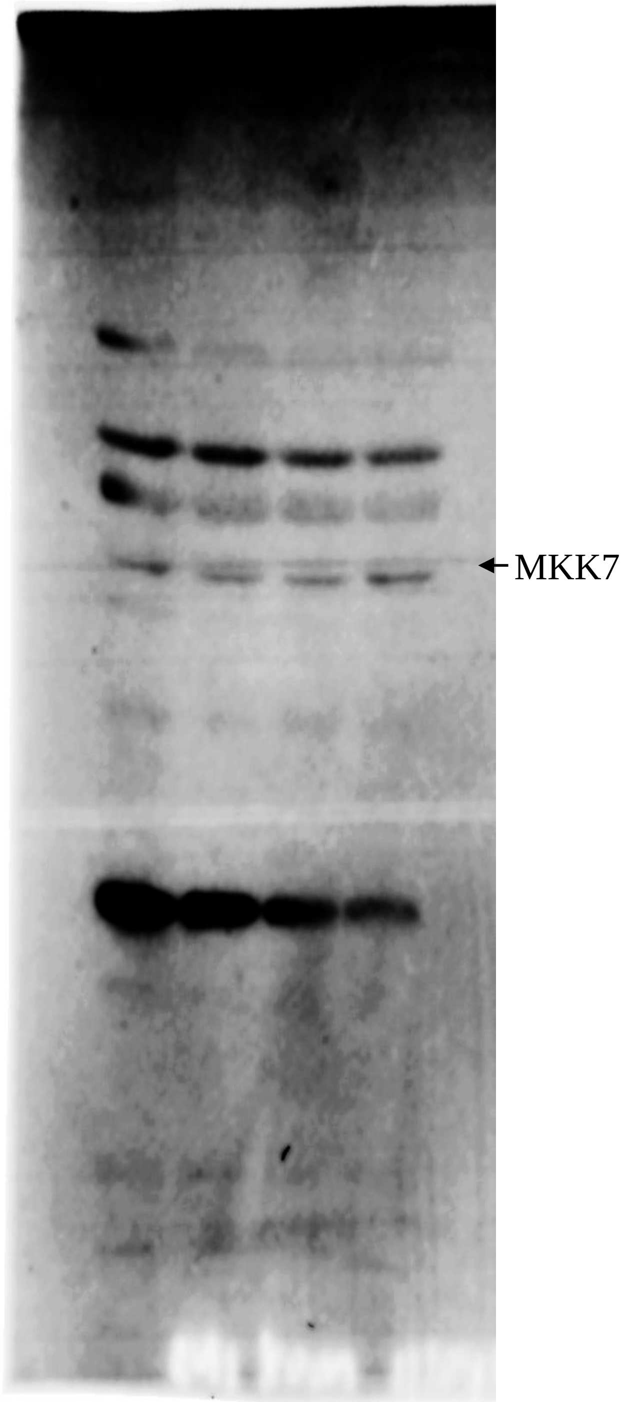
MKK7

Figure 1

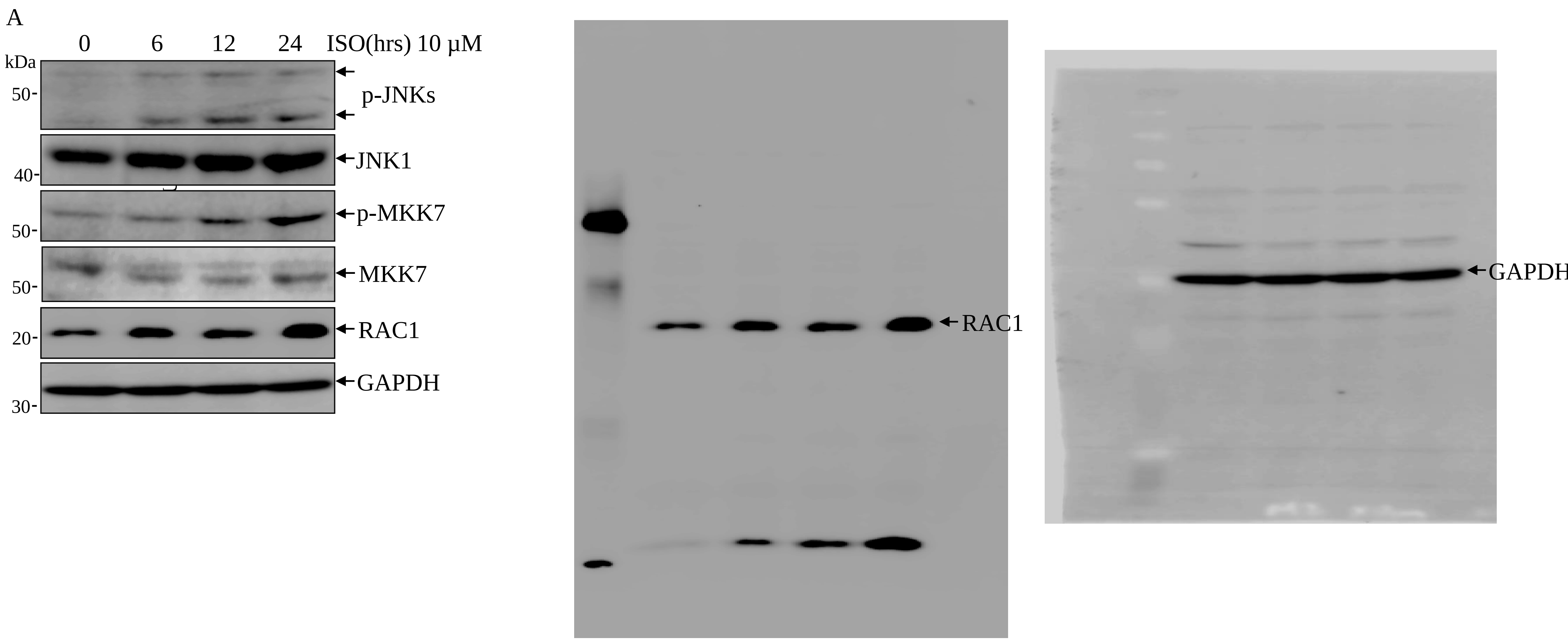
A
0
6
12
24
ISO(hrs) 10 μM
kDa
50-
p-JNKs
JNK1
40-
p-MKK7
50-
MKK7
50-
RAC1
20-
GAPDH
30-
RAC1
GAPDH

Figure 1

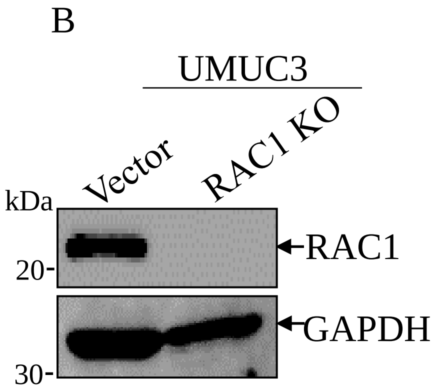

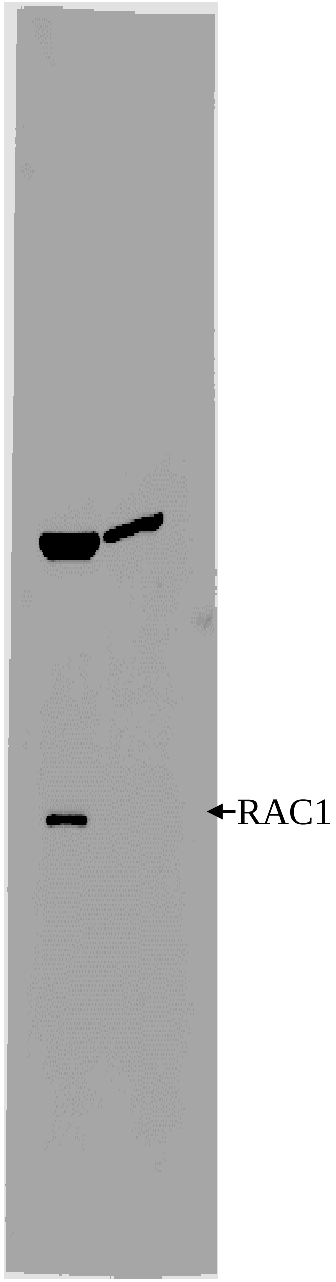

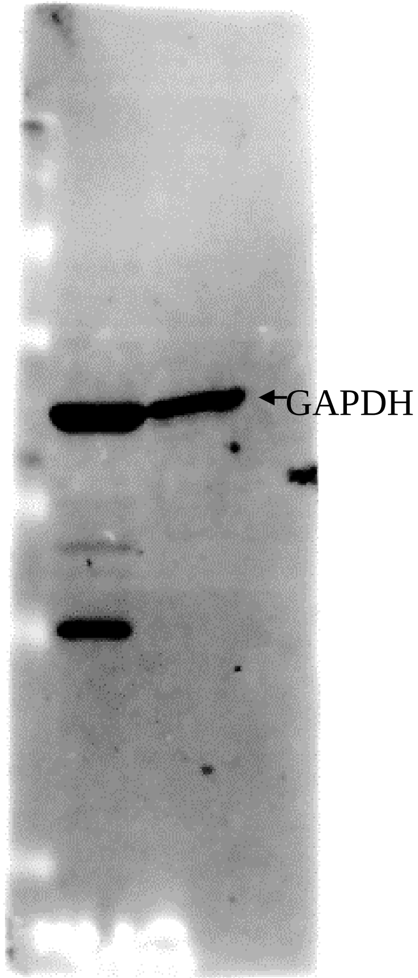

Figure 1

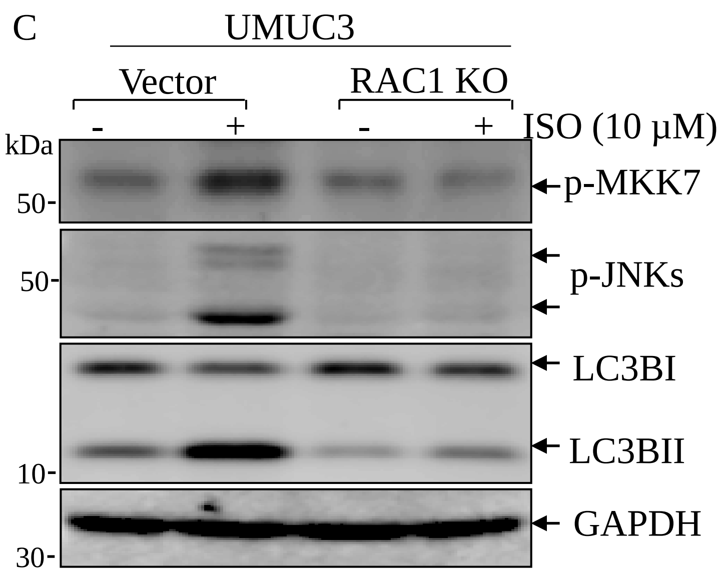
C
UMUC3
Vector
RAC1 KO
- + - + ISO (10 µM)
kDa
50-
50-
10-
30-
p-MKK7
p-JNKs
LC3BI
LC3BII
GAPDH

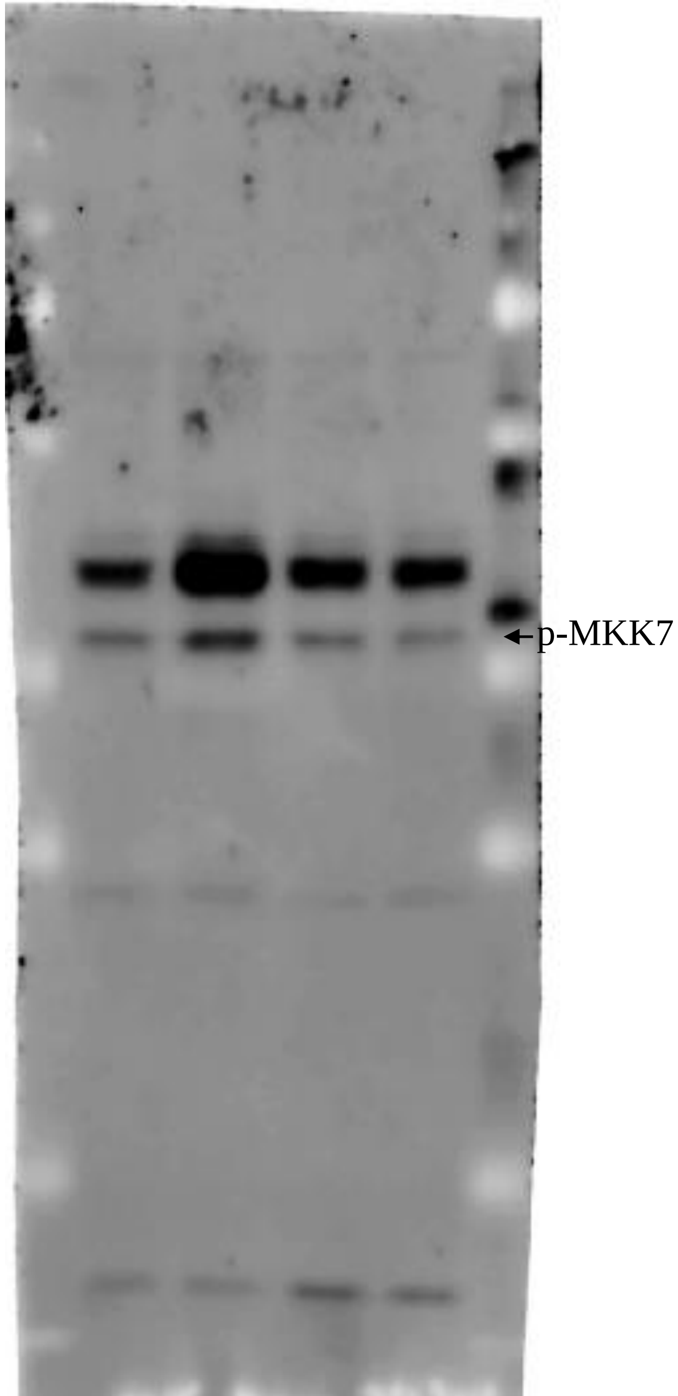
p-MKK7

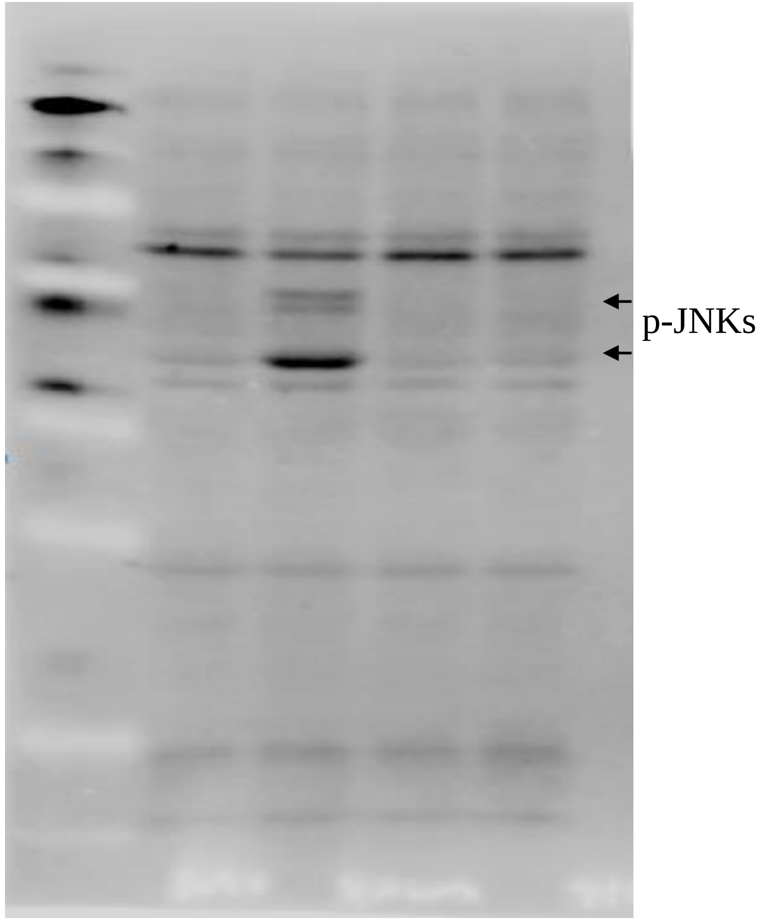
p-JNKs

Figure 1

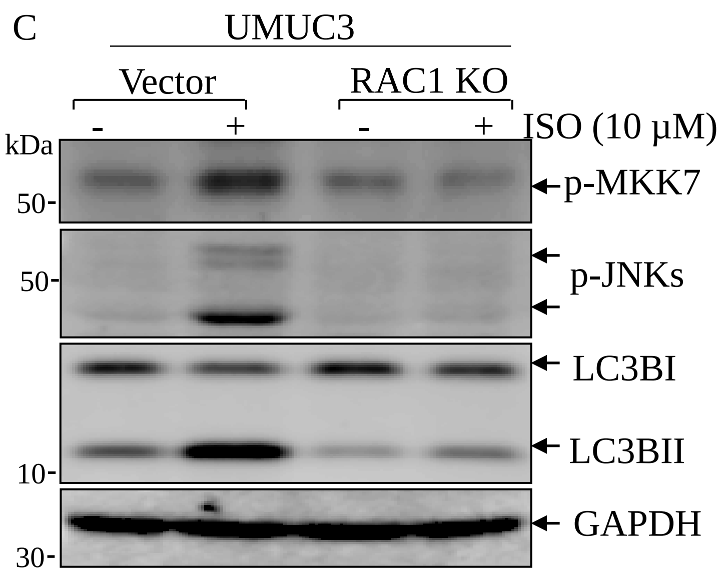
C
UMUC3
Vector
RAC1 KO
- + - + ISO (10 μM)
kDa
50-
50-
10-
30-
p-MKK7
p-JNKs
LC3BI
LC3BII
GAPDH

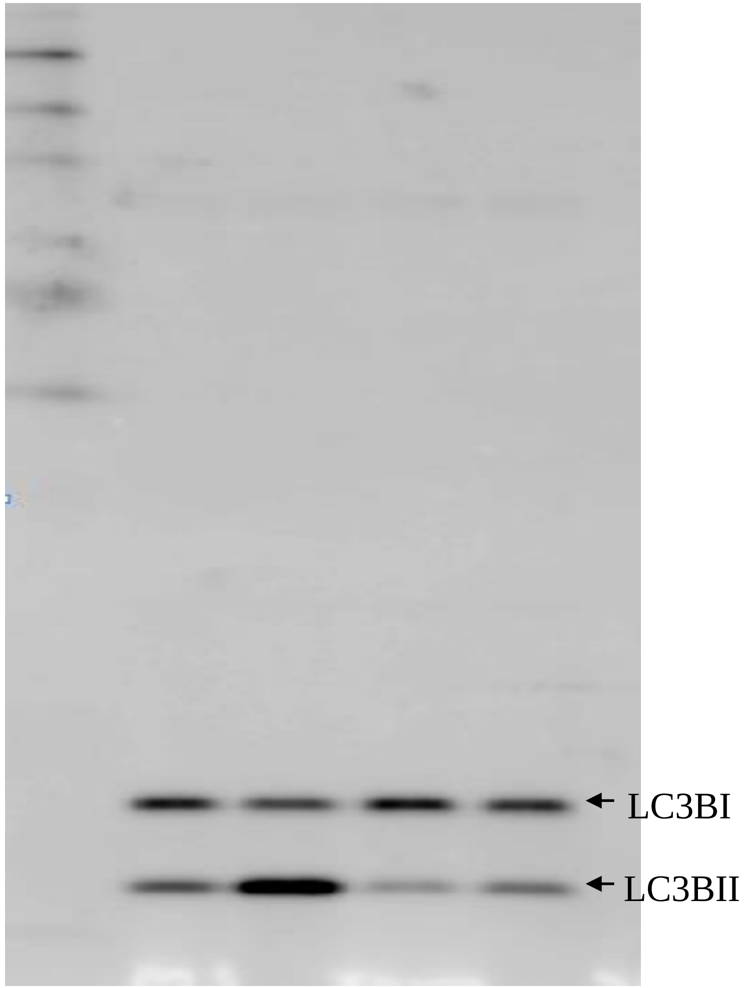
LC3BI
LC3BII

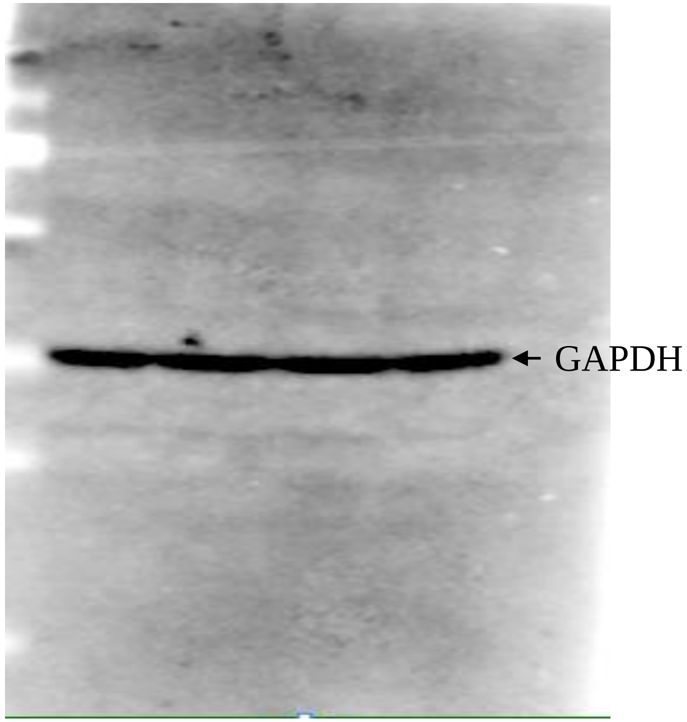
GAPDH

Figure 2

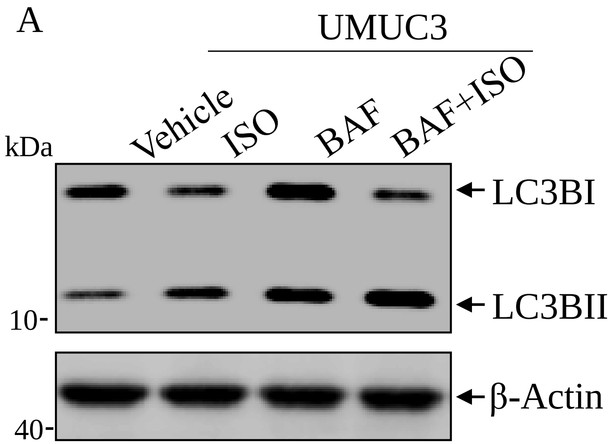
A
UMUC3
Vehicle
ISO
BAF
BAF+ISO
kDa
LC3BI
LC3BII
10-
β-Actin
40-

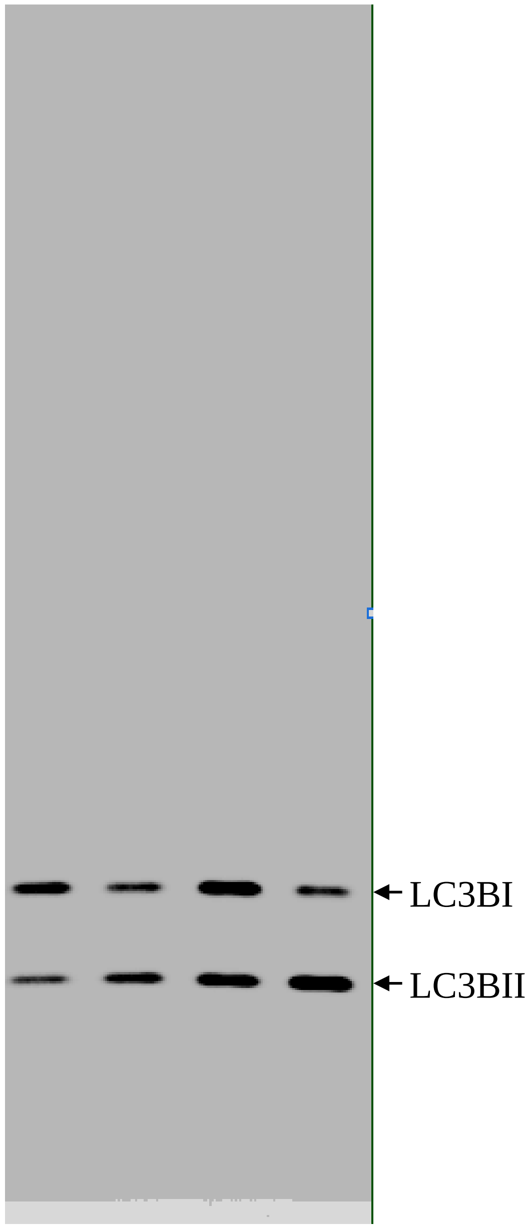
LC3BI
LC3BII

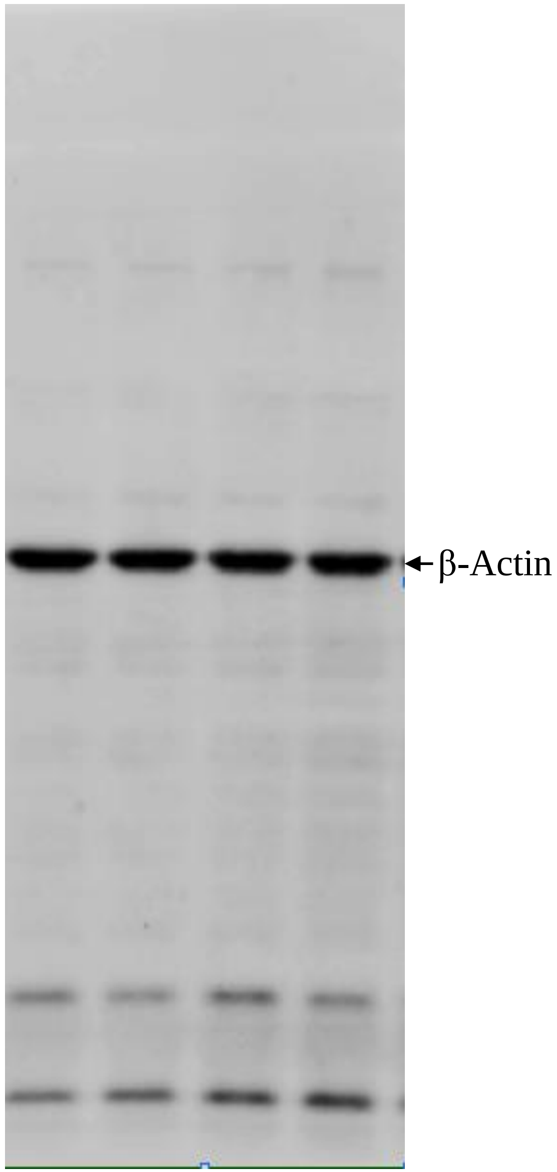
β-Actin

Figure 2

D

UMUC3

Nonsense shh*SESN2*

kDa - + - + ISO (10 µM)

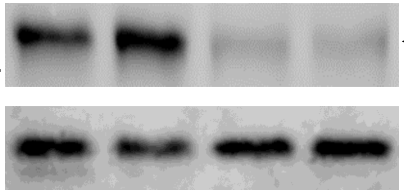

SESN2

β-Actin

50-

40-

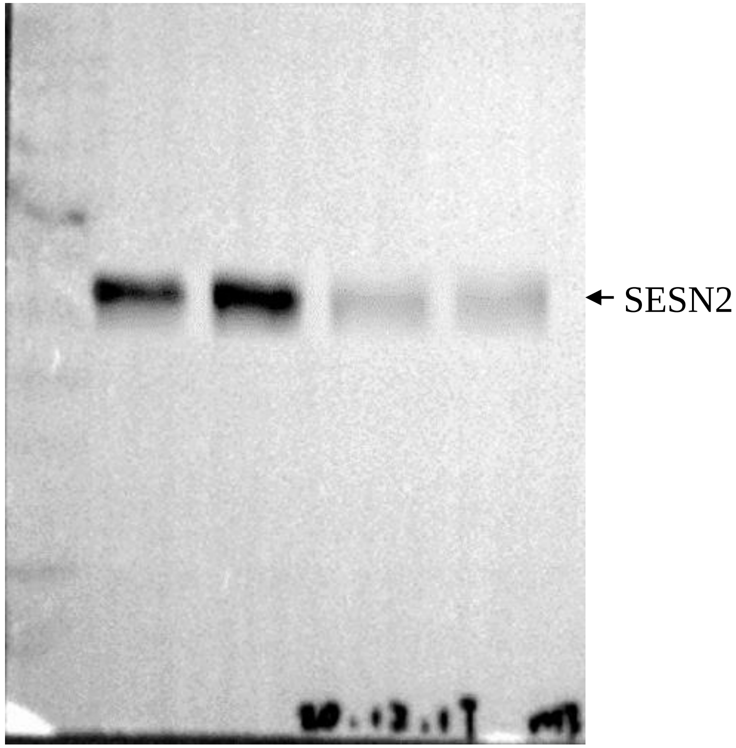

SESN2

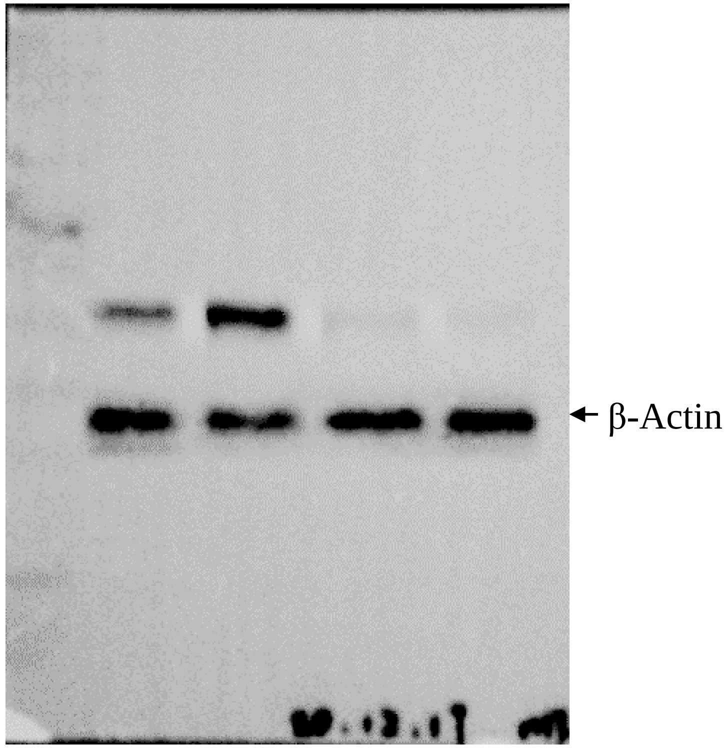

β-Actin

Figure 3

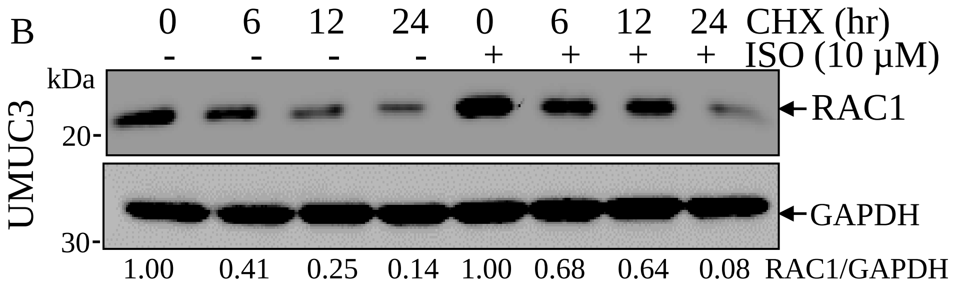
B
0 6 12 24 0 6 12 24 CHX (hr)
- - - - + + + + ISO (10 μM)
kDa
UMUC3
20-
30-
RAC1
GAPDH
1.00 0.41 0.25 0.14 1.00 0.68 0.64 0.08 RAC1/GAPDH

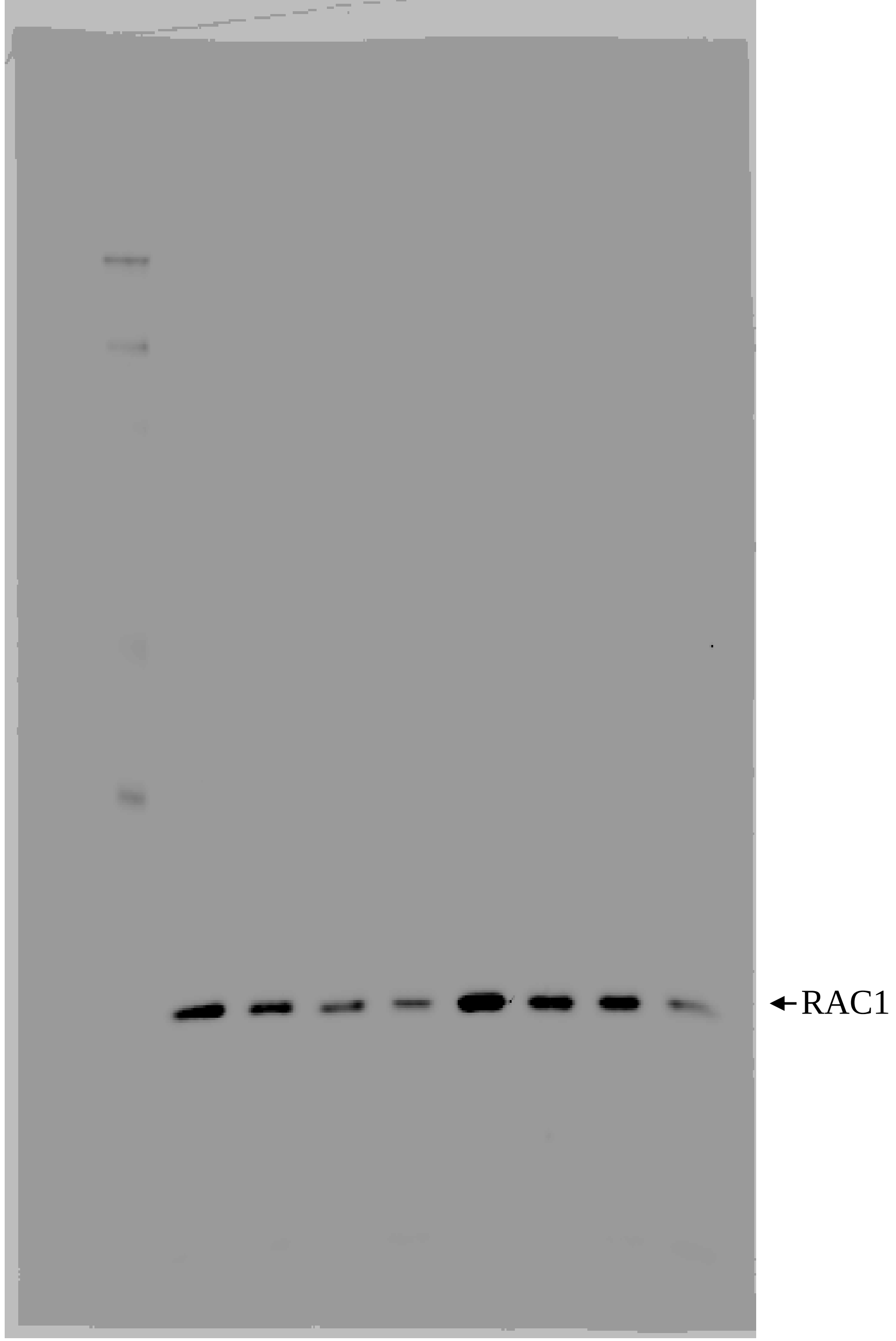
RAC1

Figure 3

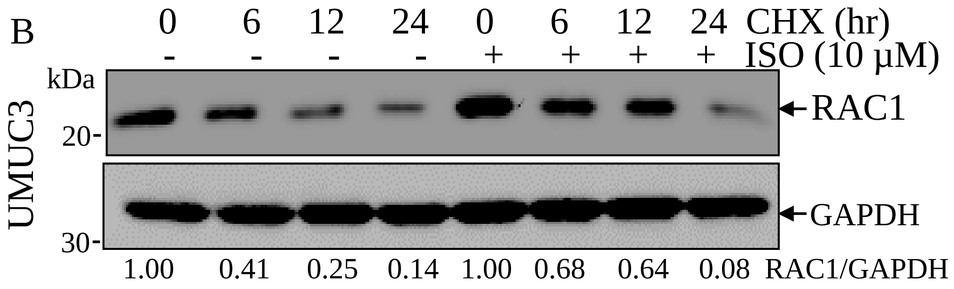

B
0 6 12 24 0 6 12 24 CHX (hr)
- - - - + + + + ISO (10 µM)
kDa
RAC1
20-
UMUC3
GAPDH
30-
1.00 0.41 0.25 0.14 1.00 0.68 0.64 0.08 RAC1/GAPDH

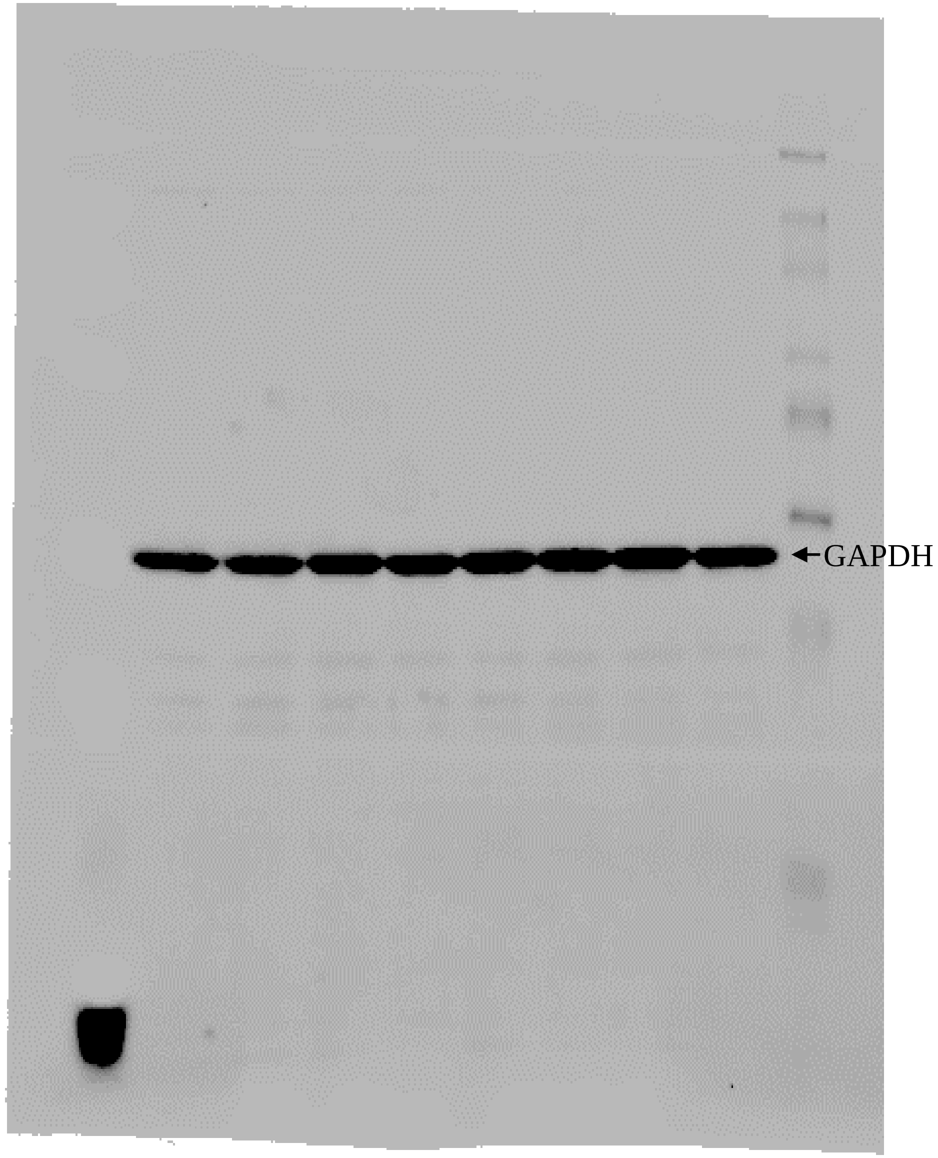

GAPDH

Figure 3

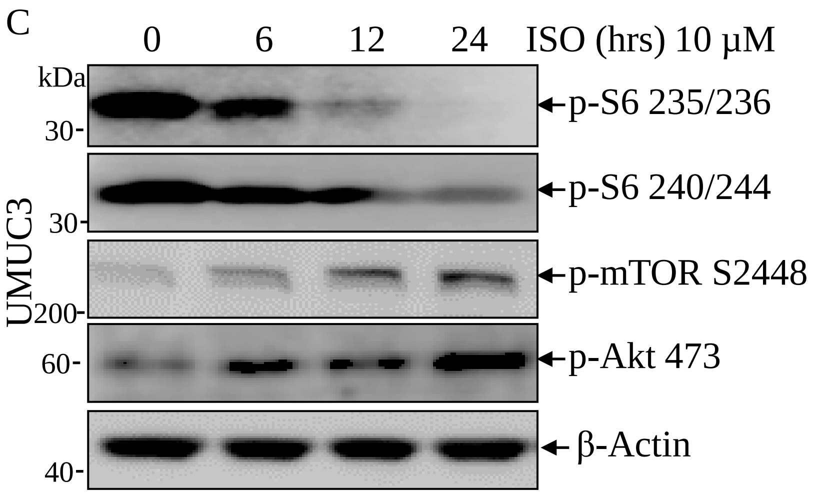
C
0
6
12
24
ISO (hrs) 10 μM
kDa
30-
30-
200-
60-
40-
UMUC3
p-S6 235/236
p-S6 240/244
p-mTOR S2448
p-Akt 473
β-Actin

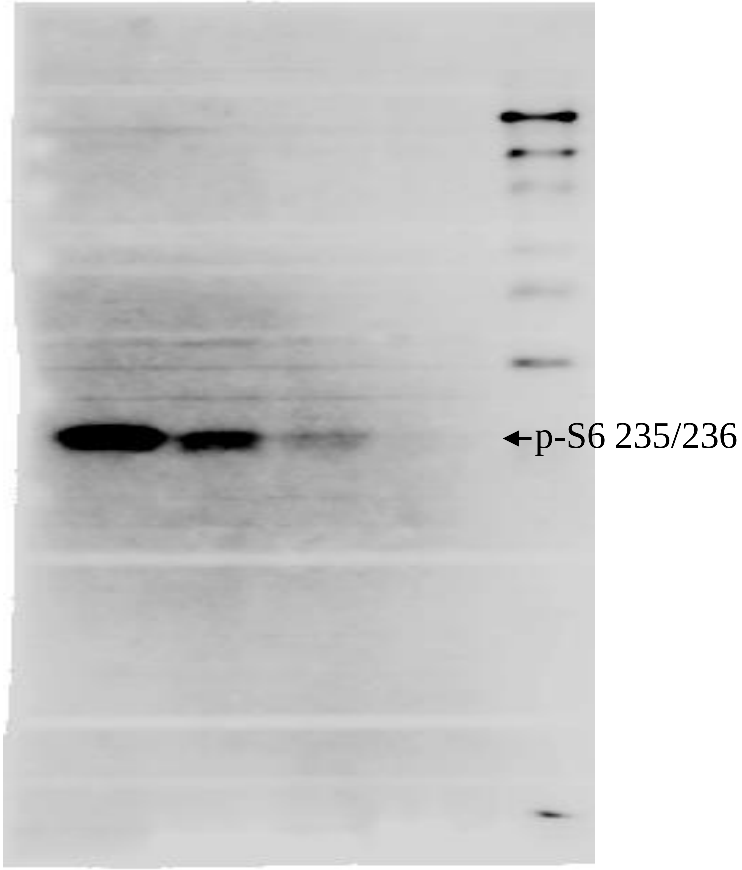
p-S6 235/236

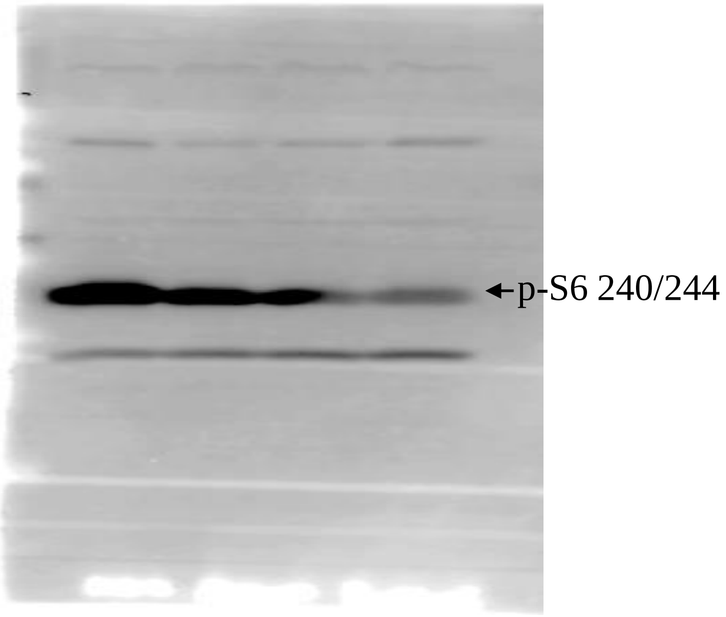
p-S6 240/244

Figure 3

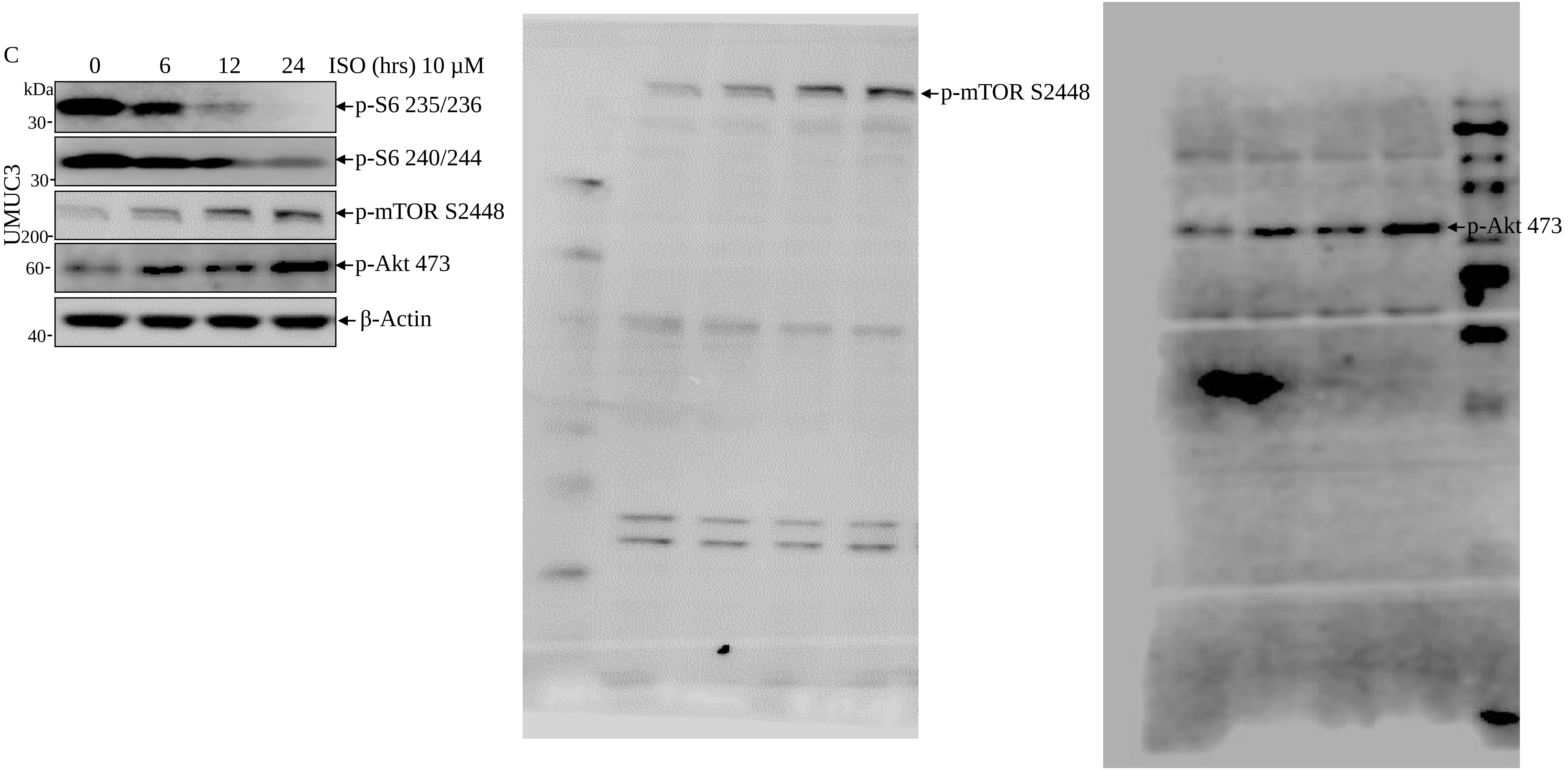
C
0
6
12
24
ISO (hrs) 10 μM
kDa
30-
30-
200-
60-
40-
UMUC3
p-S6 235/236
p-S6 240/244
p-mTOR S2448
p-Akt 473
β-Actin
p-mTOR S2448
p-Akt 473

Figure 3

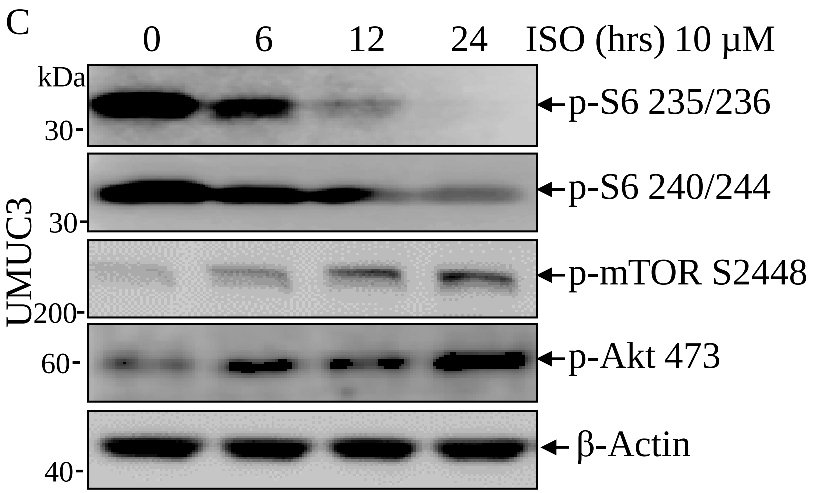
C
0
6
12
24
ISO (hrs) 10 µM
kDa
30
p-S6 235/236
30
p-S6 240/244
UMUC3
200
p-mTOR S2448
60
p-Akt 473
40
β-Actin

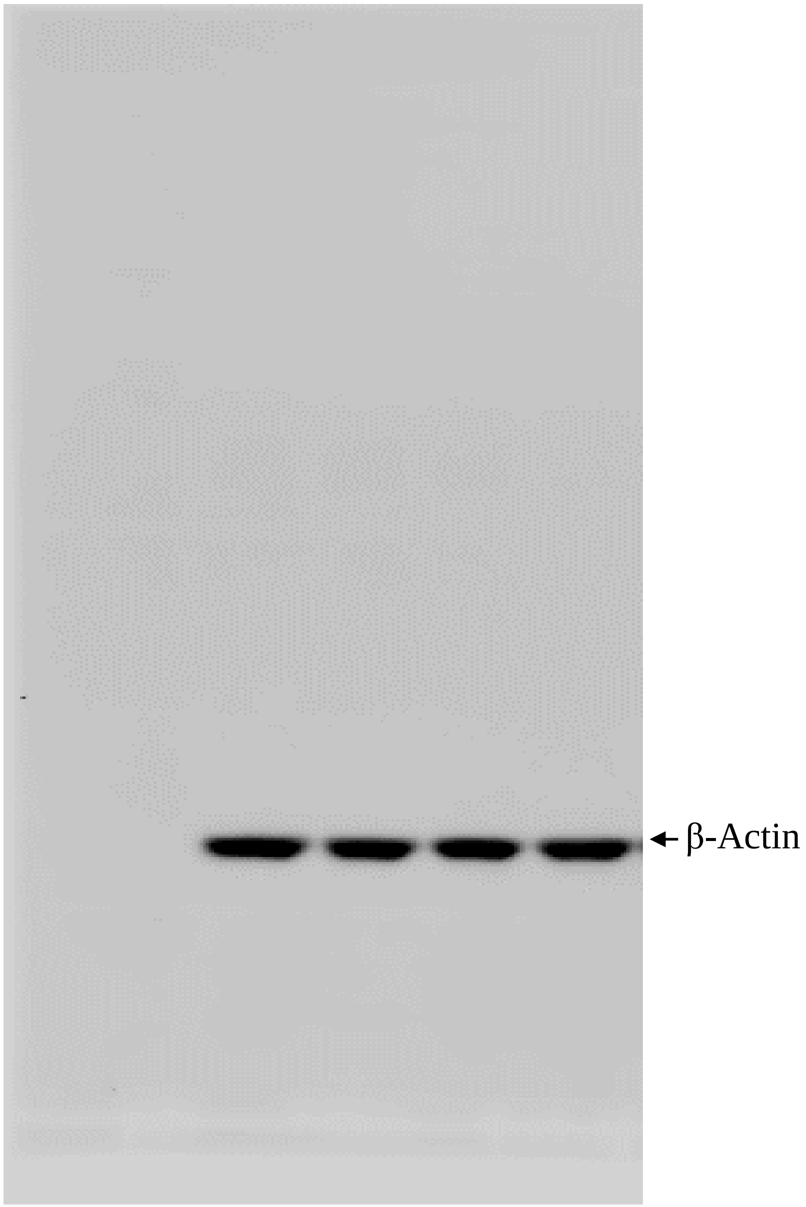
β-Actin

Figure 3

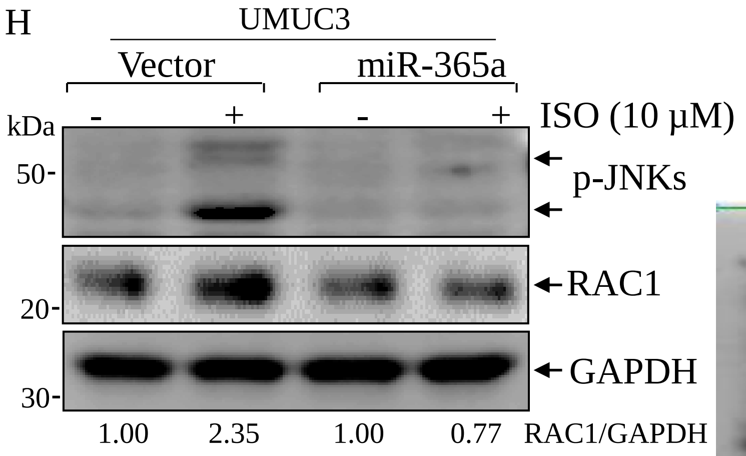
H
UMUC3
Vector
miR-365a
- + - +
ISO (10 μM)
kDa
50-
20-
30-
p-JNKs
RAC1
GAPDH
1.00
2.35
1.00
0.77
RAC1/GAPDH

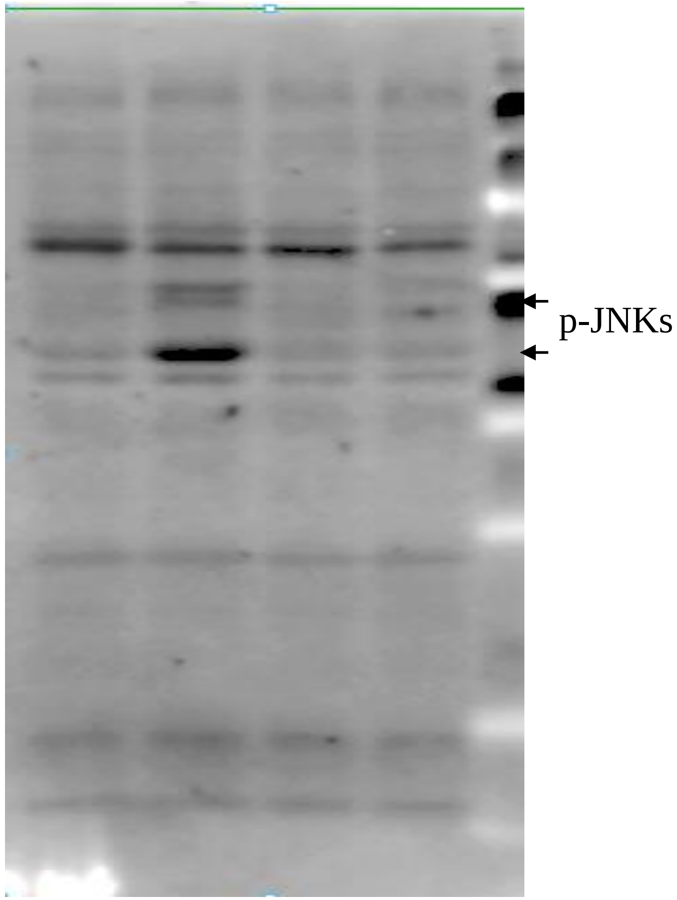
p-JNKs

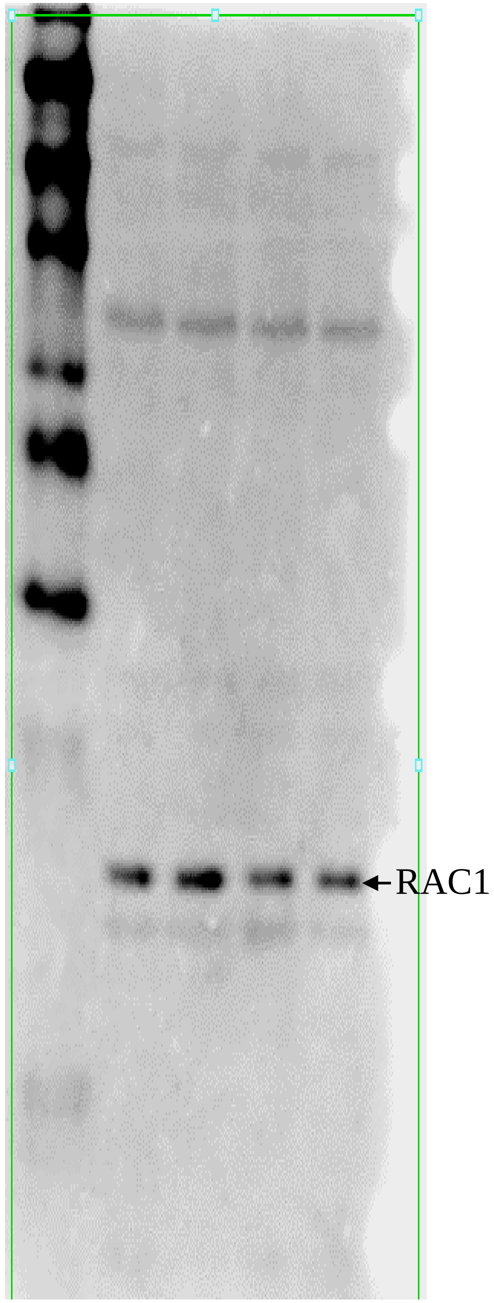
RAC1

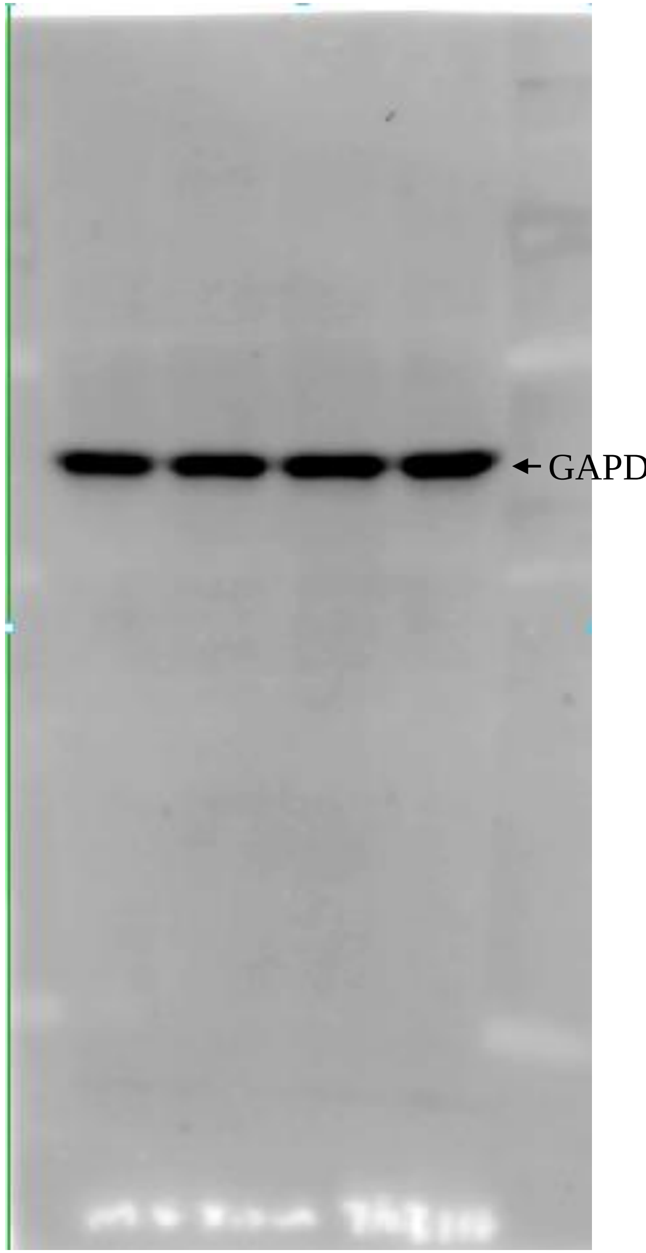
GAPD

Figure 4

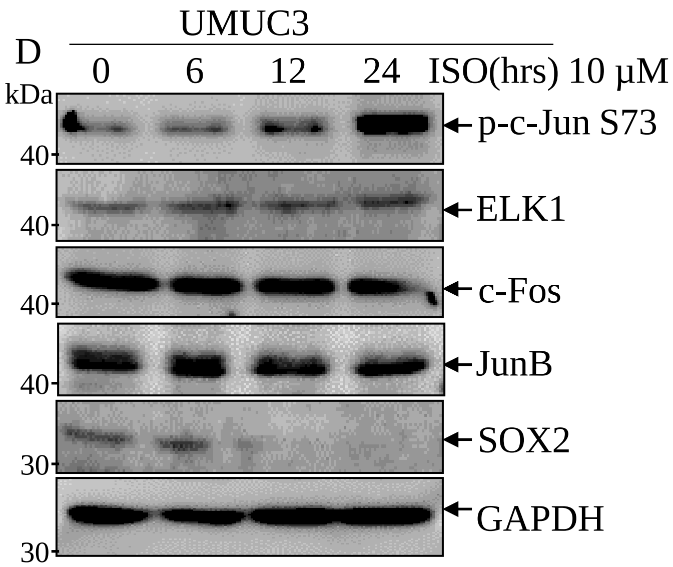
D
UMUC3
0
6
12
24
ISO(hrs) 10 μM
kDa
40
40
40
40
30
30
p-c-Jun S73
ELK1
c-Fos
JunB
SOX2
GAPDH

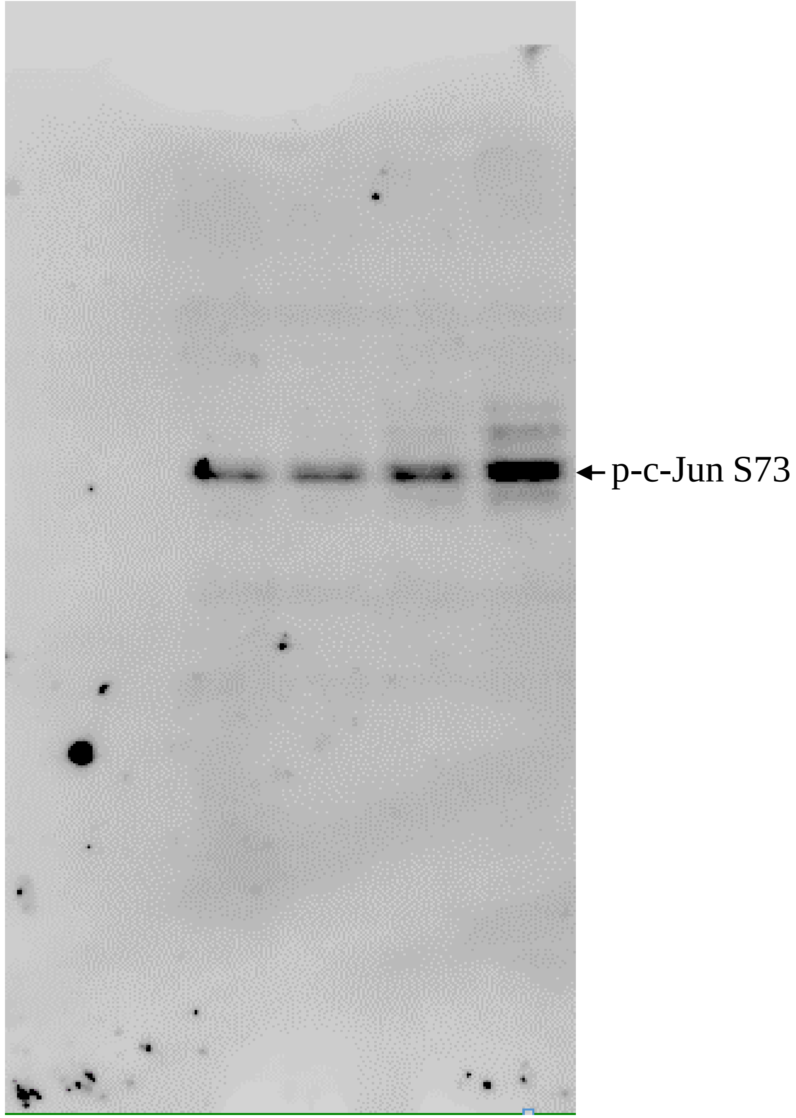
p-c-Jun S73

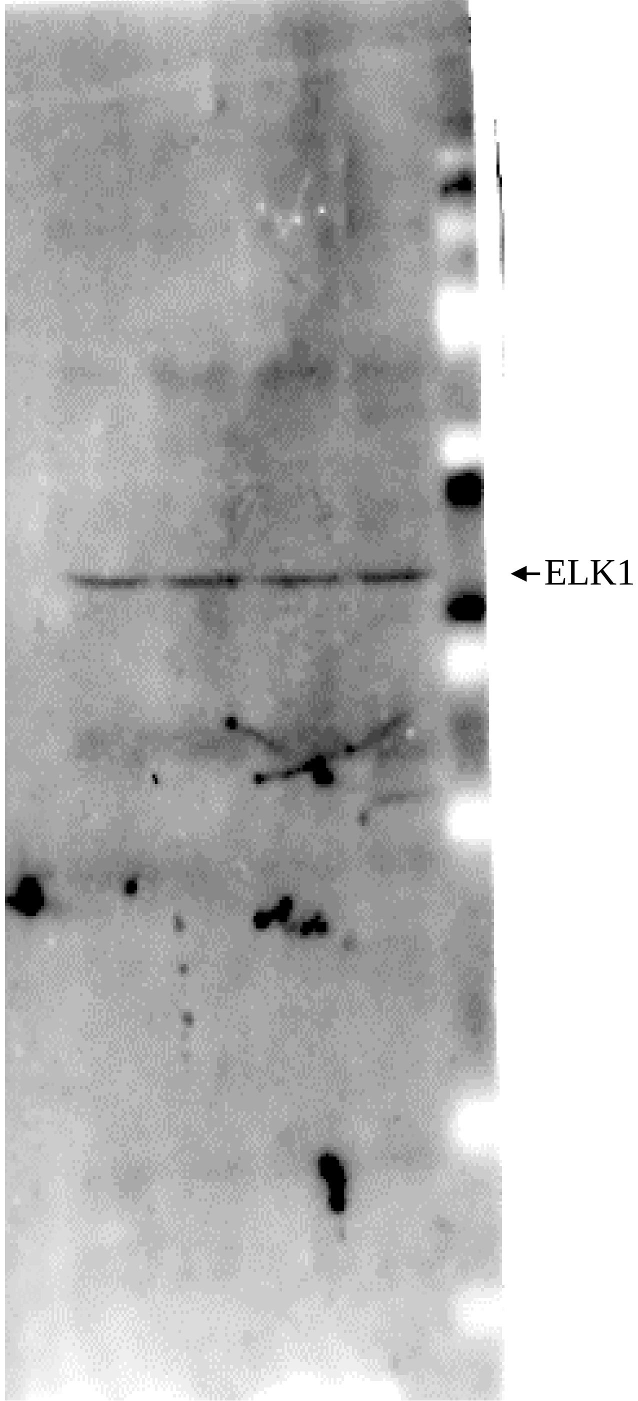
ELK1

Figure 4

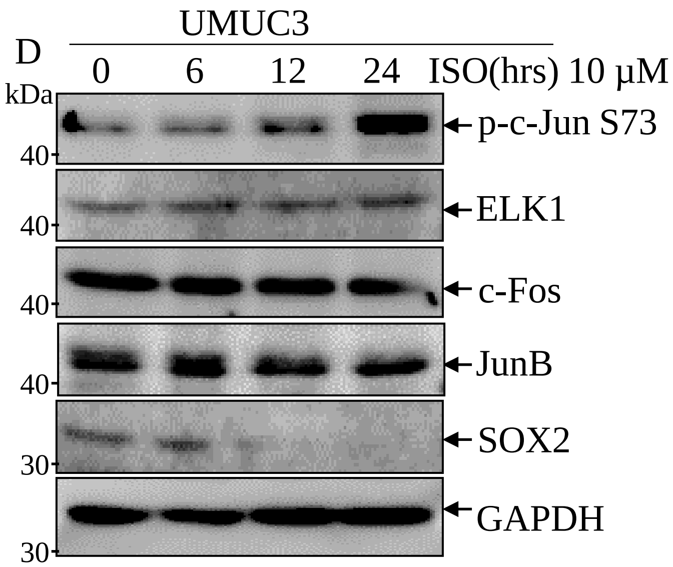
D
UMUC3
0
6
12
24
ISO(hrs) 10 μM
kDa
p-c-Jun S73
ELK1
c-Fos
JunB
SOX2
GAPDH
40
40
40
40
30
30

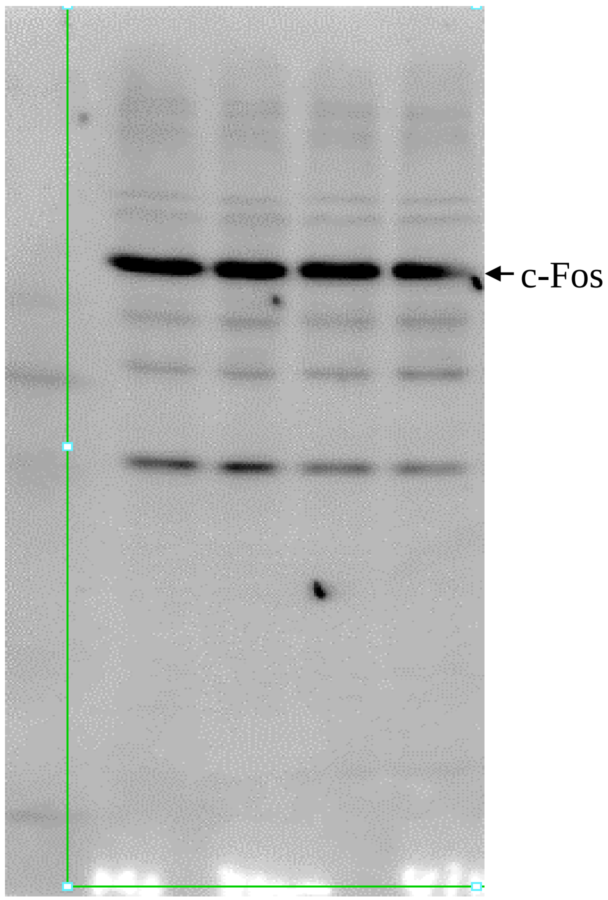
c-Fos

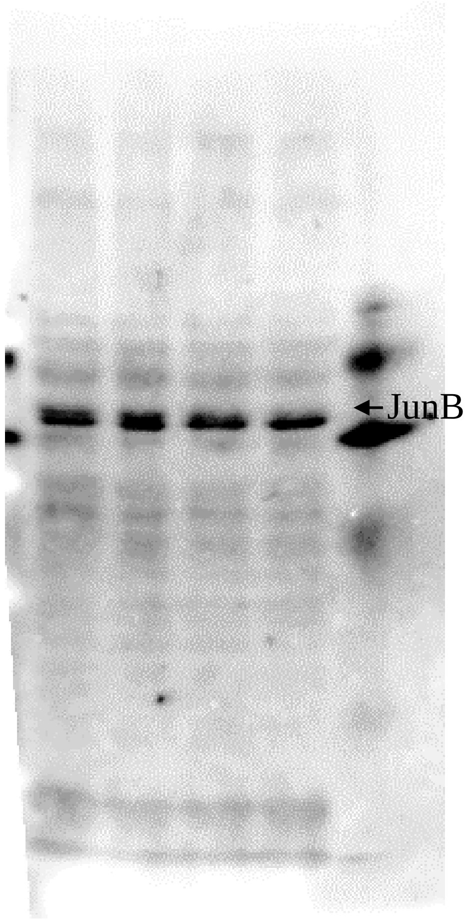
JunB

Figure 4

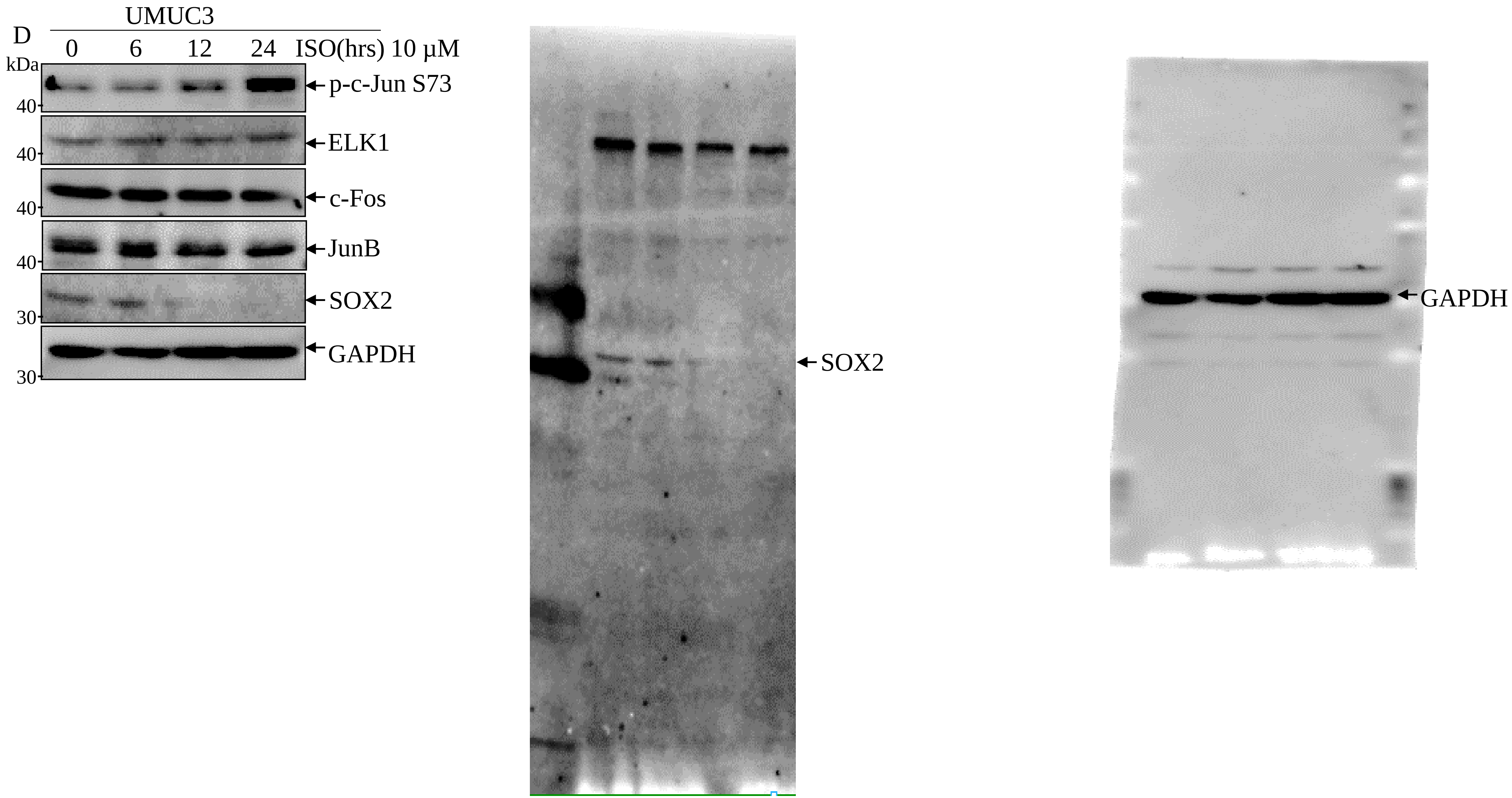

D
UMUC3
0
6
12
24
ISO(hrs) 10 μM
kDa
40
40
40
40
30
30
p-c-Jun S73
ELK1
c-Fos
JunB
SOX2
GAPDH
SOX2
GAPDH

Figure 4

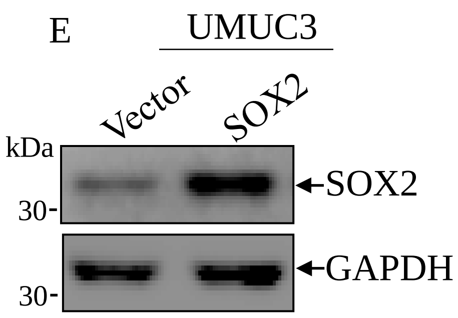

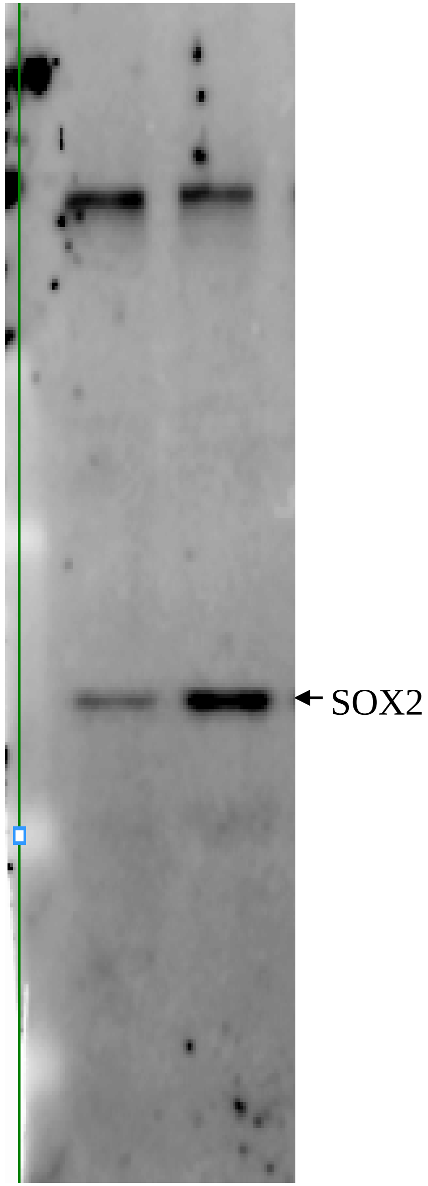

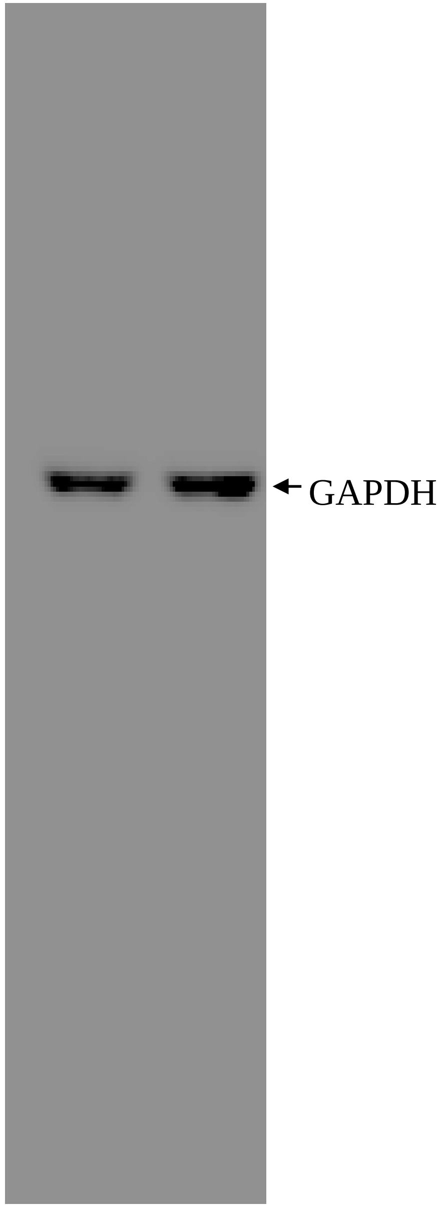

Figure 4

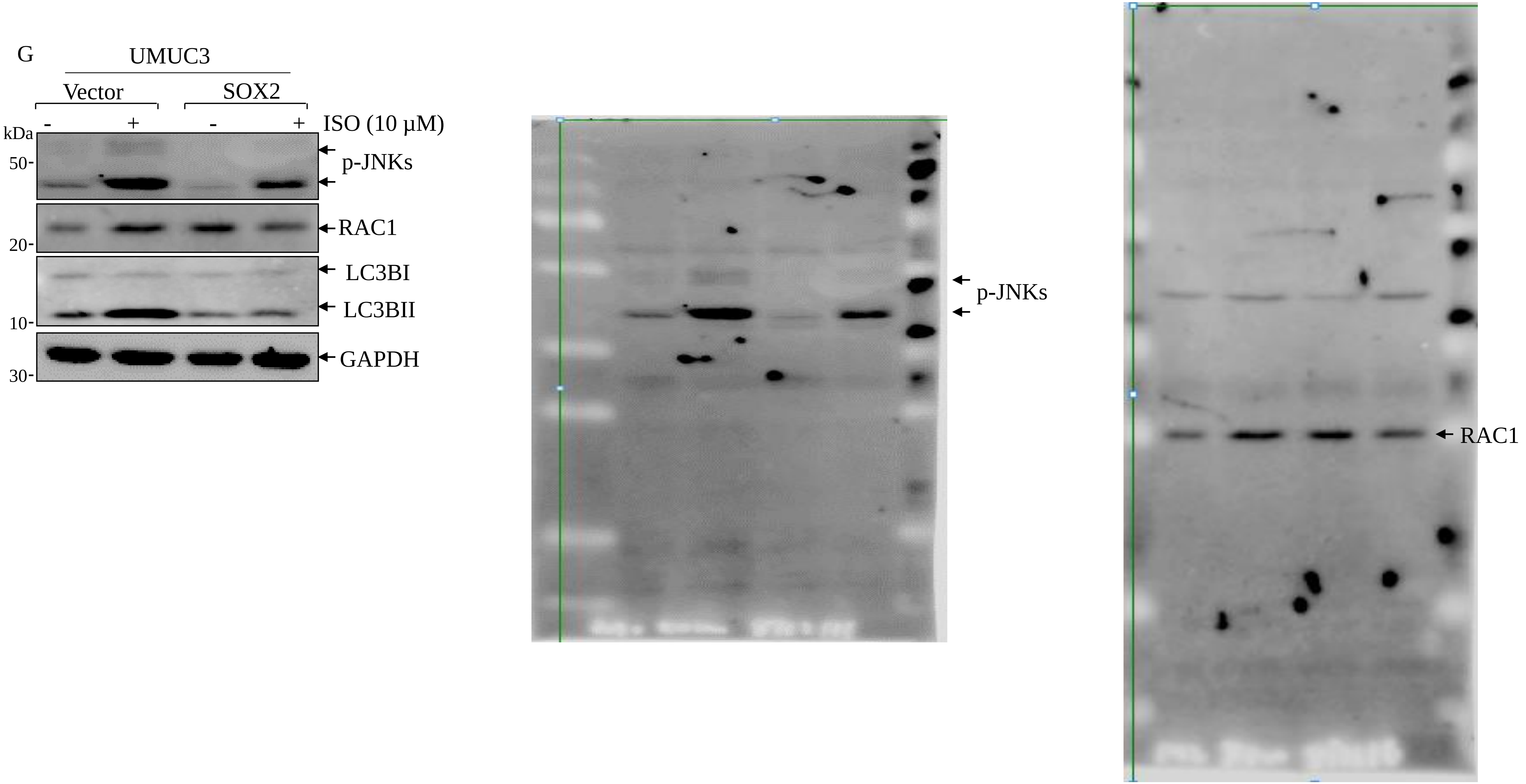
G
UMUC3
Vector
SOX2
- + - + ISO (10 μM)
kDa
50-
20-
10-
30-
p-JNKs
RAC1
LC3BI
LC3BII
GAPDH
p-JNKs
RAC1

Figure 4

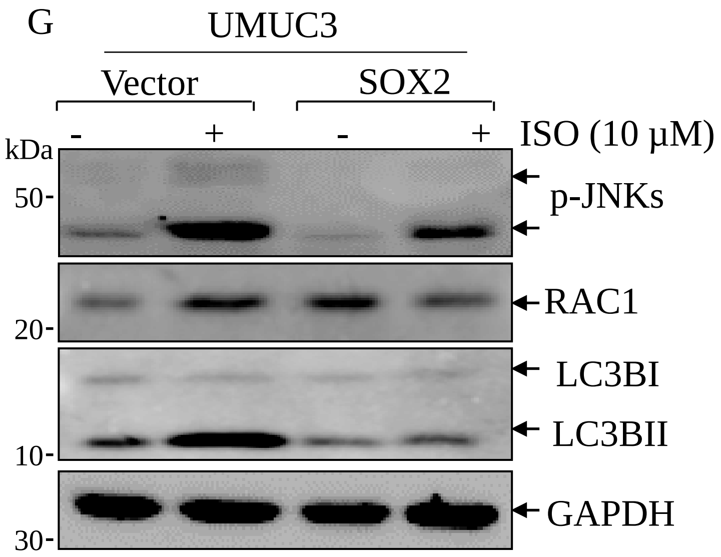
G
UMUC3
Vector
SOX2
- + - + ISO (10 μM)
kDa
50-
20-
10-
30-
p-JNKs
RAC1
LC3BI
LC3BII
GAPDH

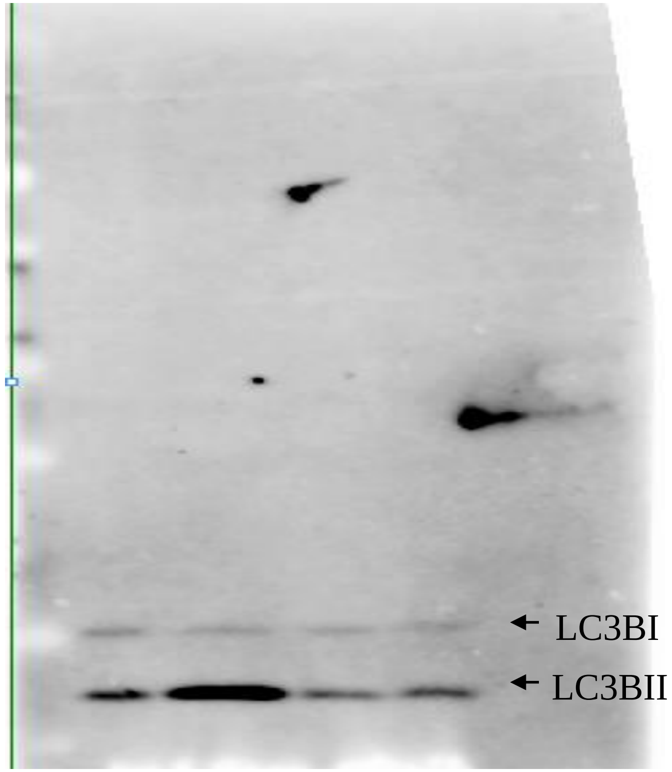
LC3BI
LC3BII

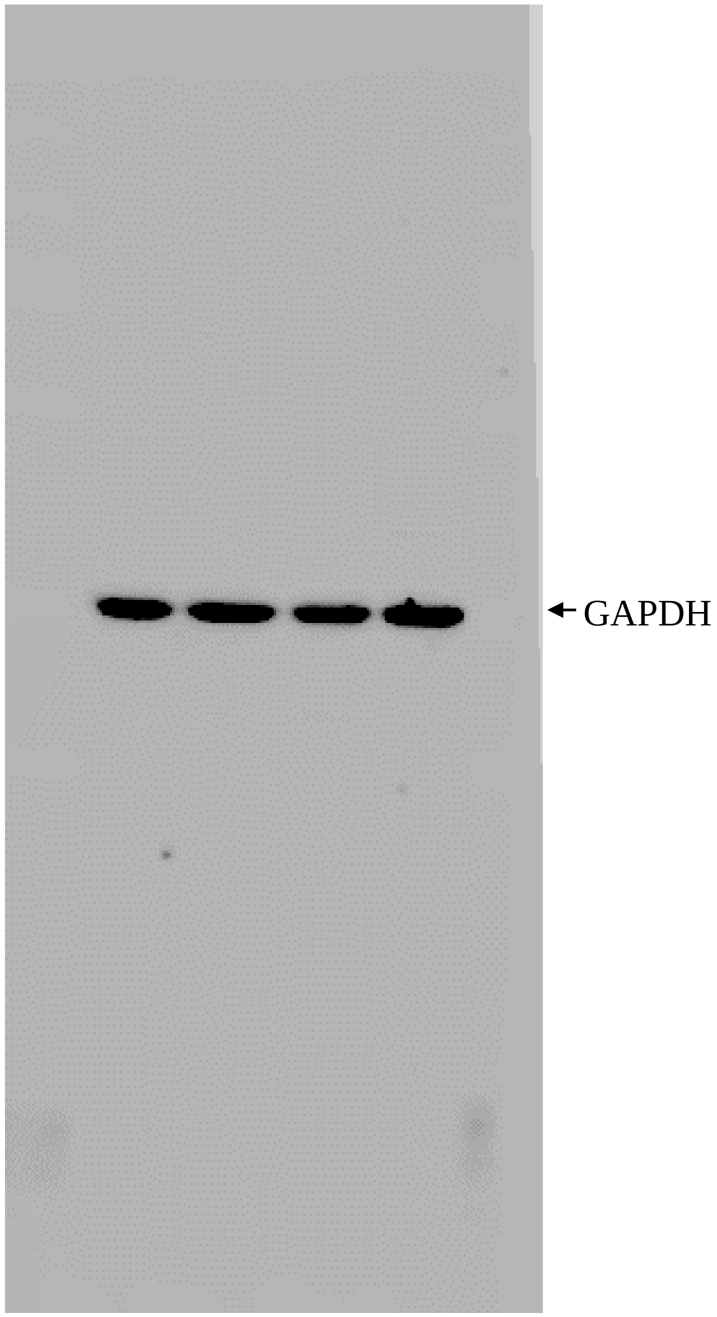
GAPDH

Figure 5

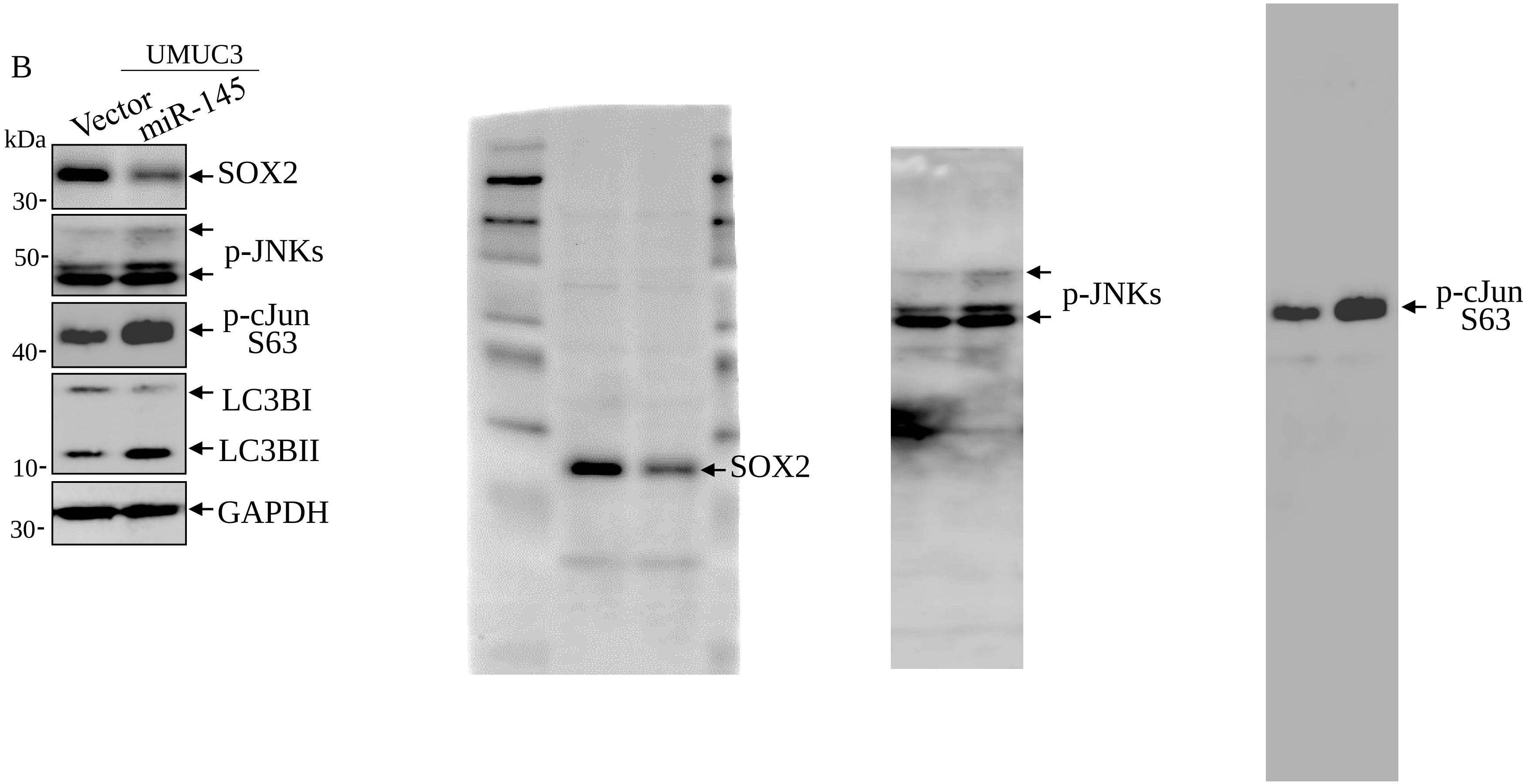
B
UMUC3
Vector
miR-145
kDa
SOX2
30-
p-JNKs
50-
p-cJun
S63
40-
LC3BI
LC3BII
10-
GAPDH
30-
SOX2
p-JNKs
p-cJun
S63

Figure 5

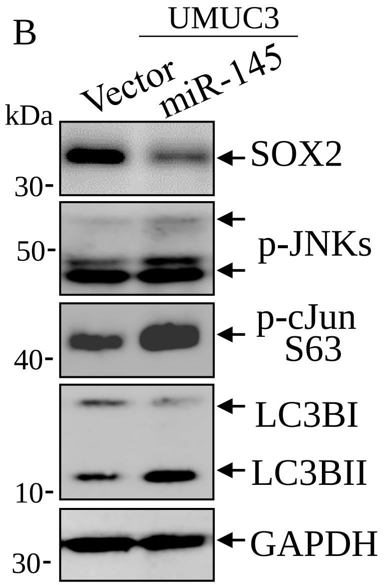
B
UMUC3
Vector
miR-145
kDa
SOX2
30-
p-JNKs
50-
p-cJun
S63
40-
LC3BI
LC3BII
10-
GAPDH
30-

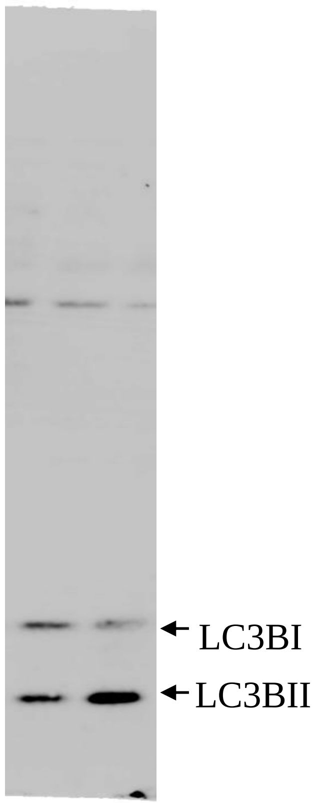
LC3BI
LC3BII

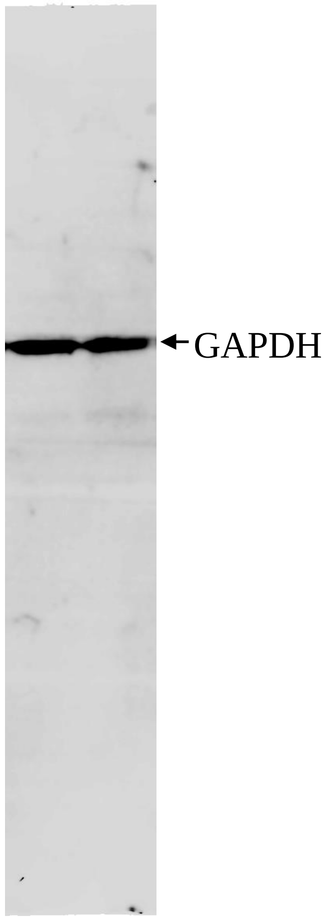
GAPDH

Figure 5

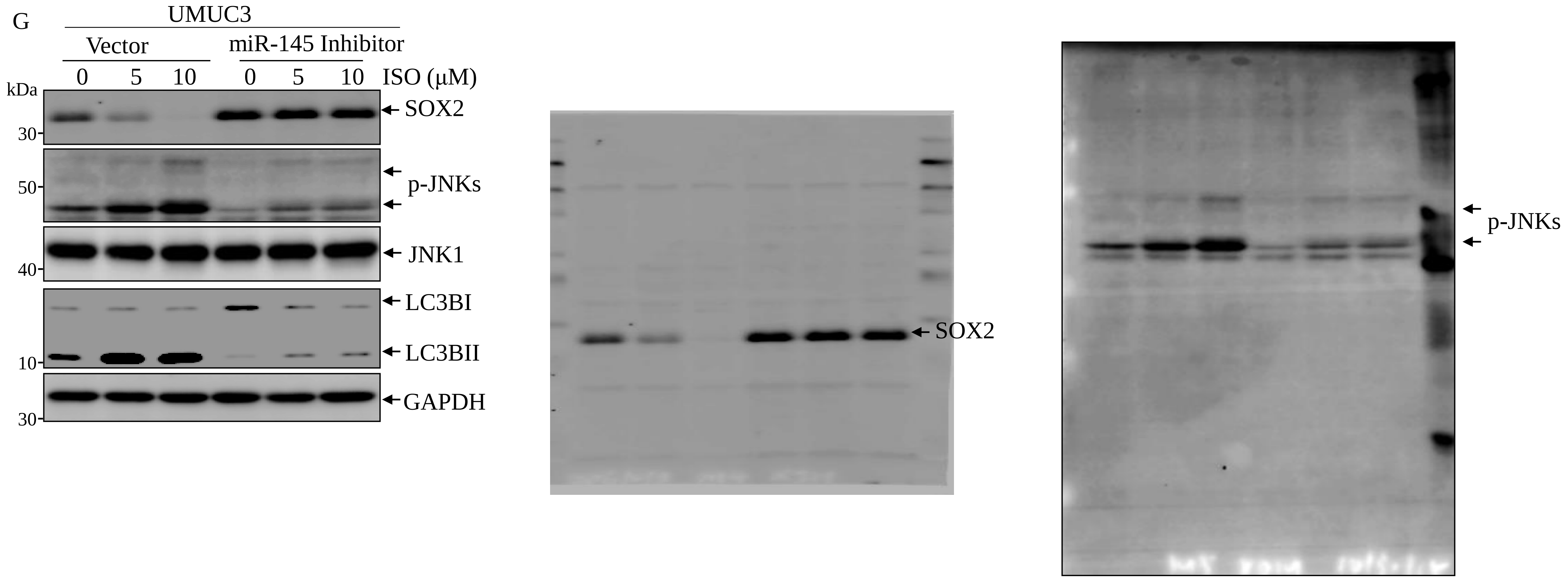
G
UMUC3
Vector
miR-145 Inhibitor
0 5 10 0 5 10 ISO (μM)
kDa
30
50
40
10
30
SOX2
p-JNKs
JNK1
LC3BI
LC3BII
GAPDH
SOX2
p-JNKs

Figure 5

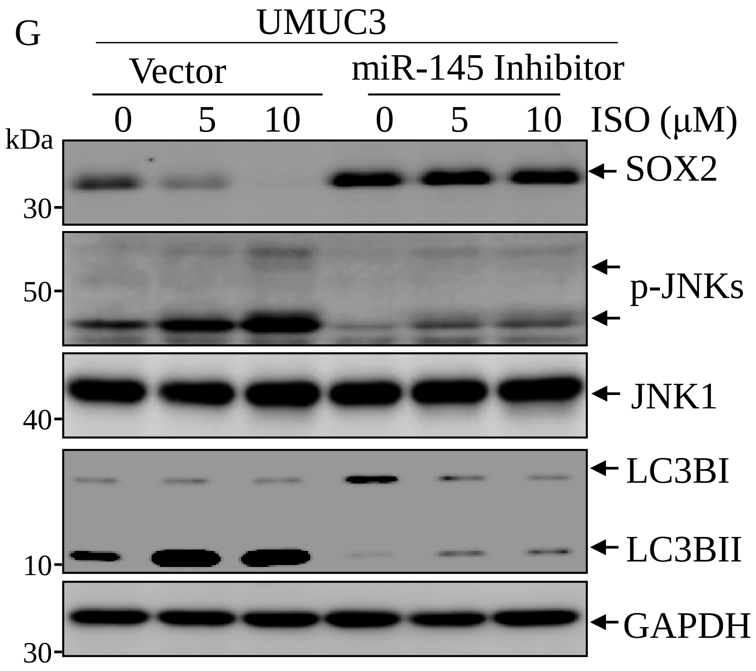
G
UMUC3
Vector
miR-145 Inhibitor
0
5
10
0
5
10
ISO (μM)
kDa
SOX2
30
p-JNKs
50
JNK1
40
LC3BI
LC3BII
10
GAPDH
30

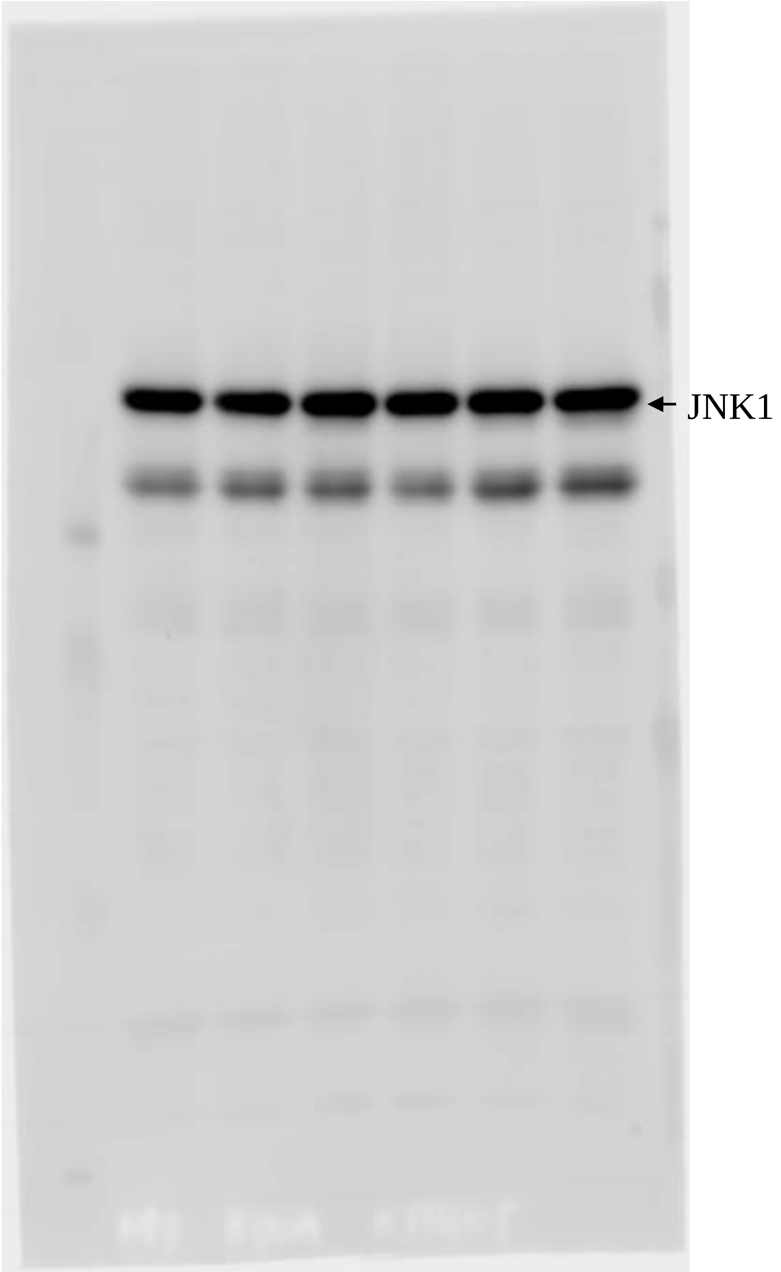
JNK1

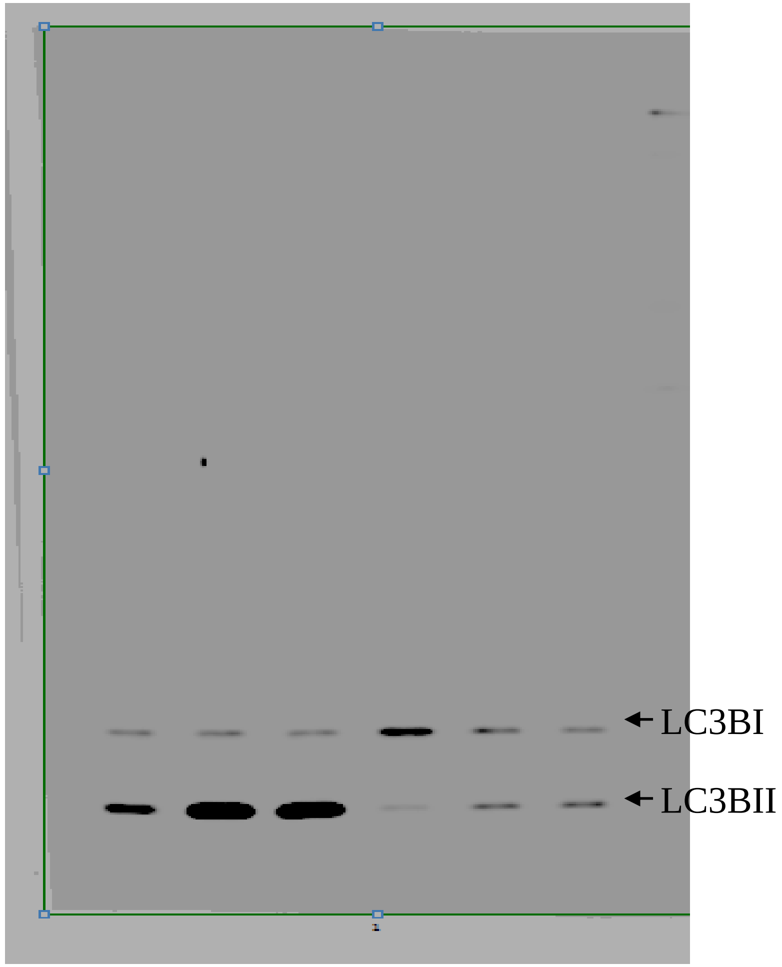
LC3BI
LC3BII

Figure 5

G

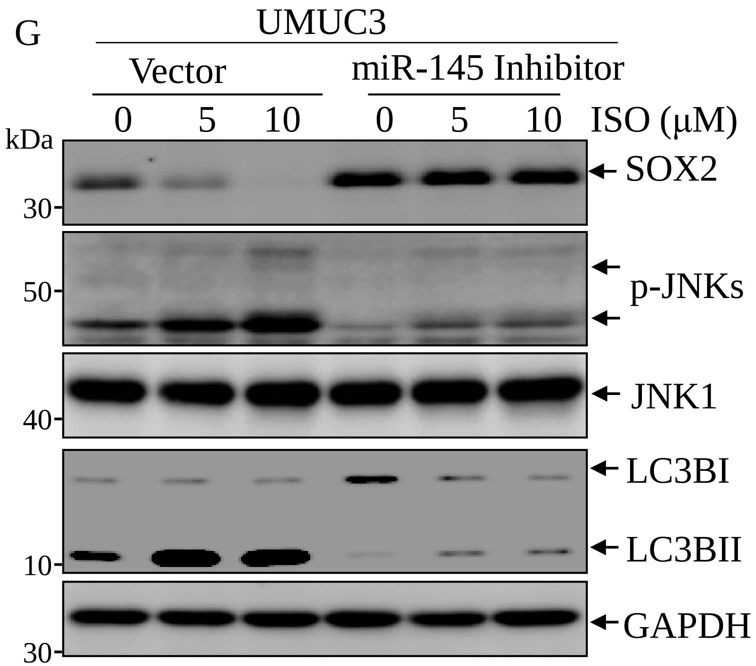
UMUC3
Vector
miR-145 Inhibitor
0
5
10
0
5
10
ISO (μM)
kDa
30
SOX2
50
p-JNKs
40
JNK1
LC3BI
LC3BII
10
30
GAPDH

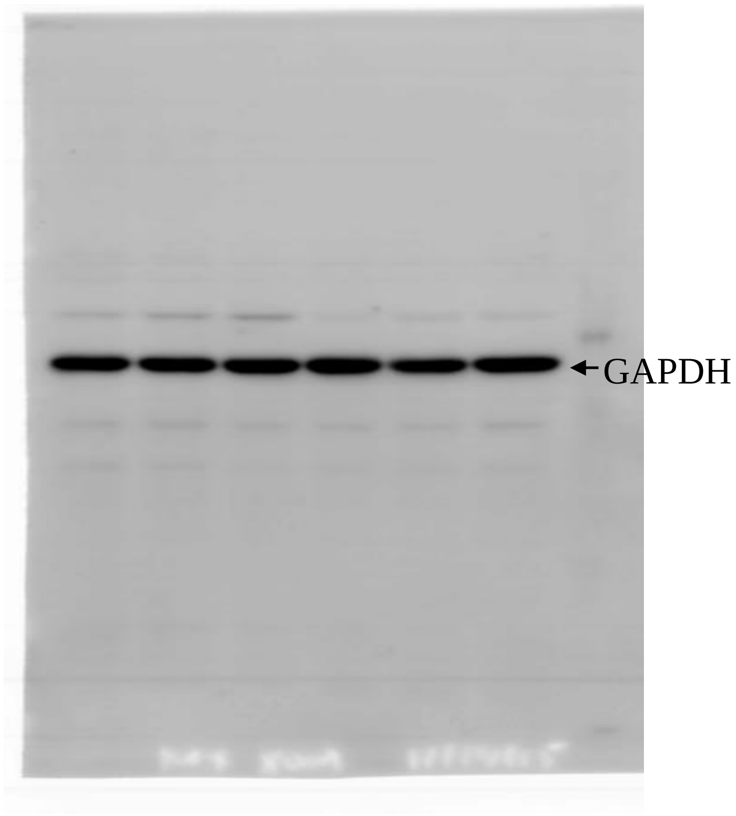
GAPDH

Figure 6

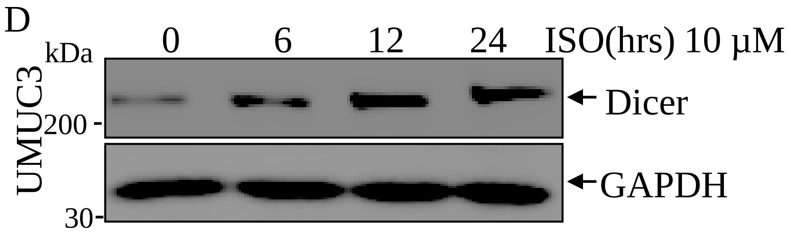
D
kDa
0
6
12
24
ISO(hrs) 10 μM
UMUC3
200
30
Dicer
GAPDH

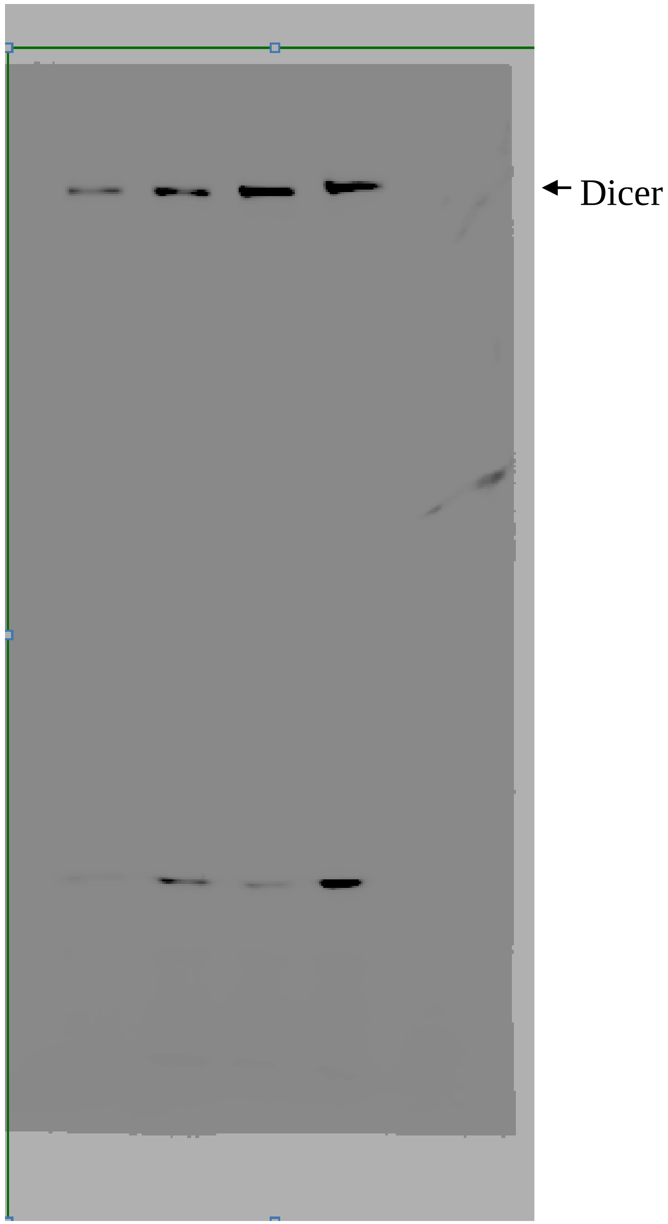
Dicer

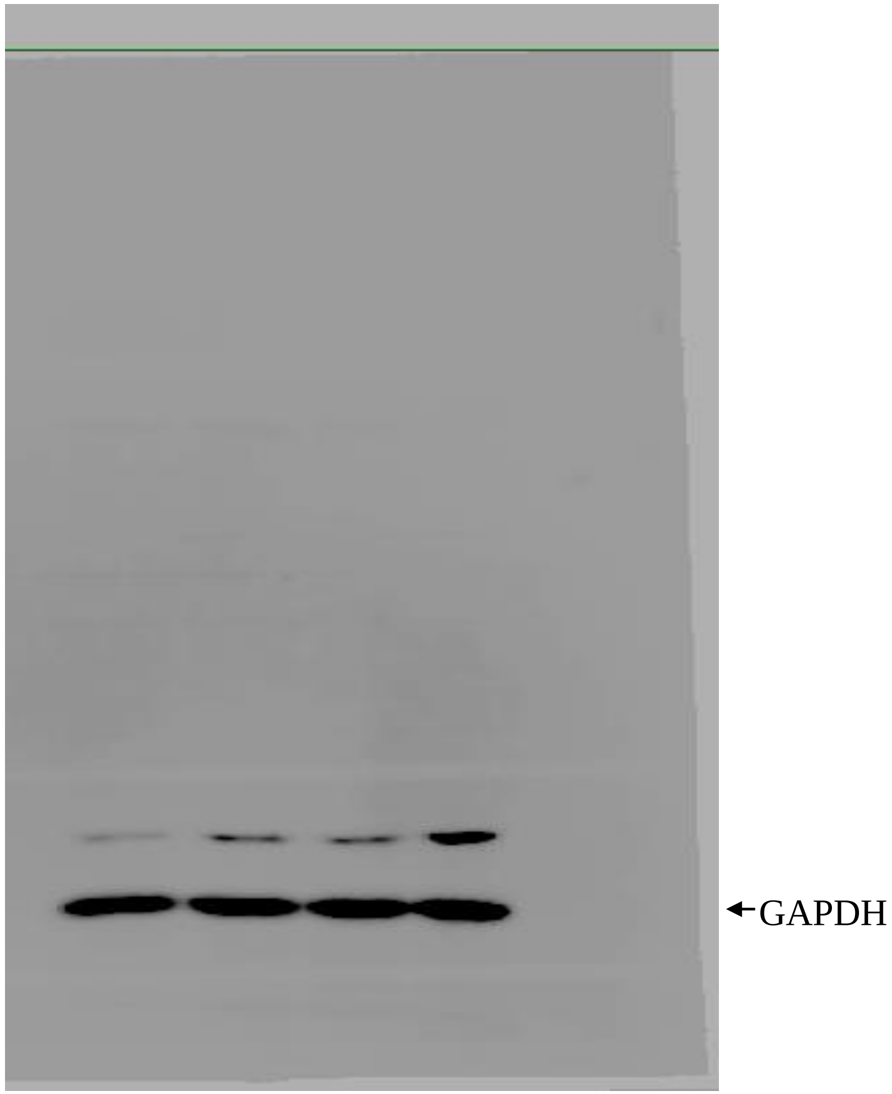
GAPDH

Figure 6

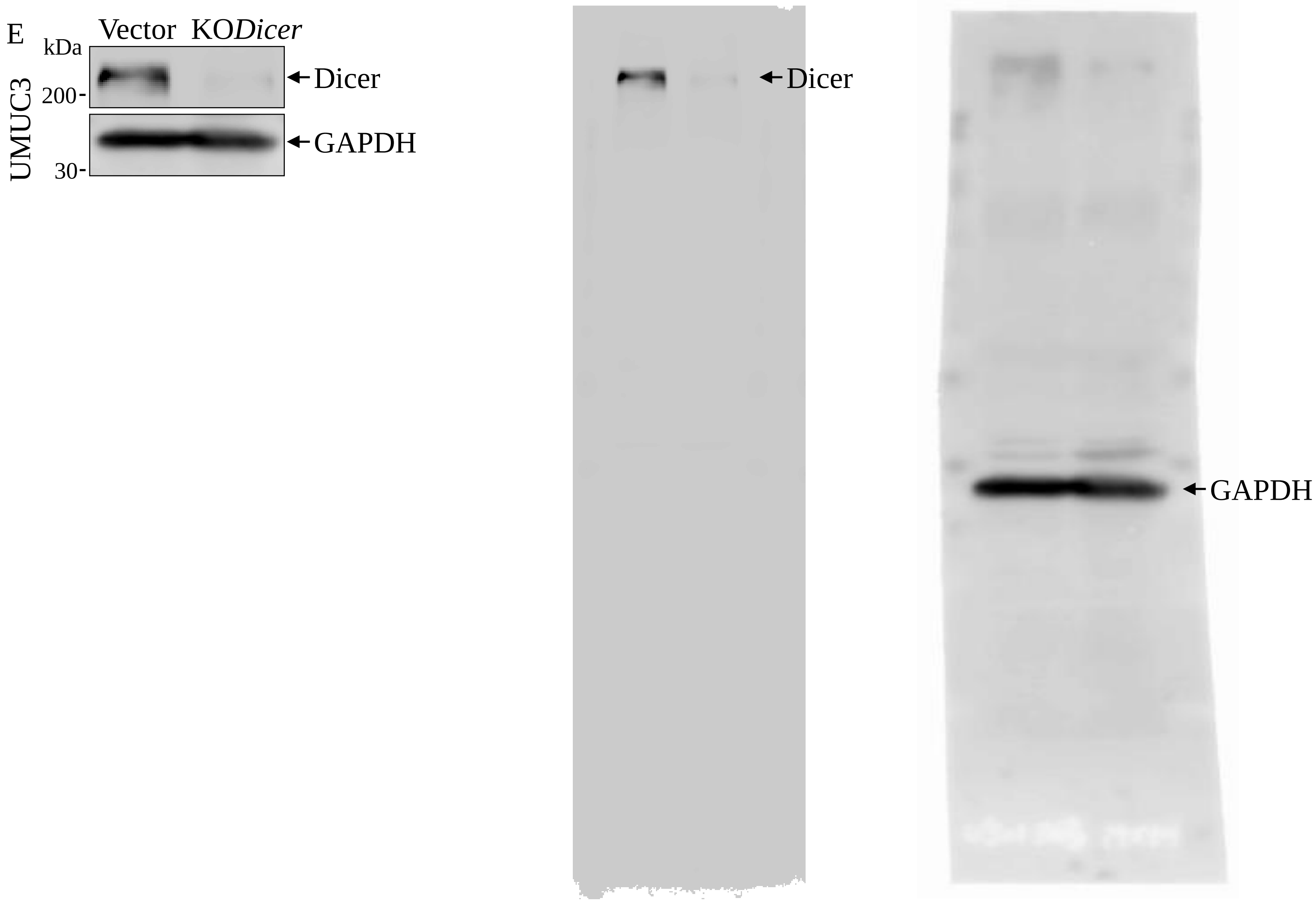

Figure 6

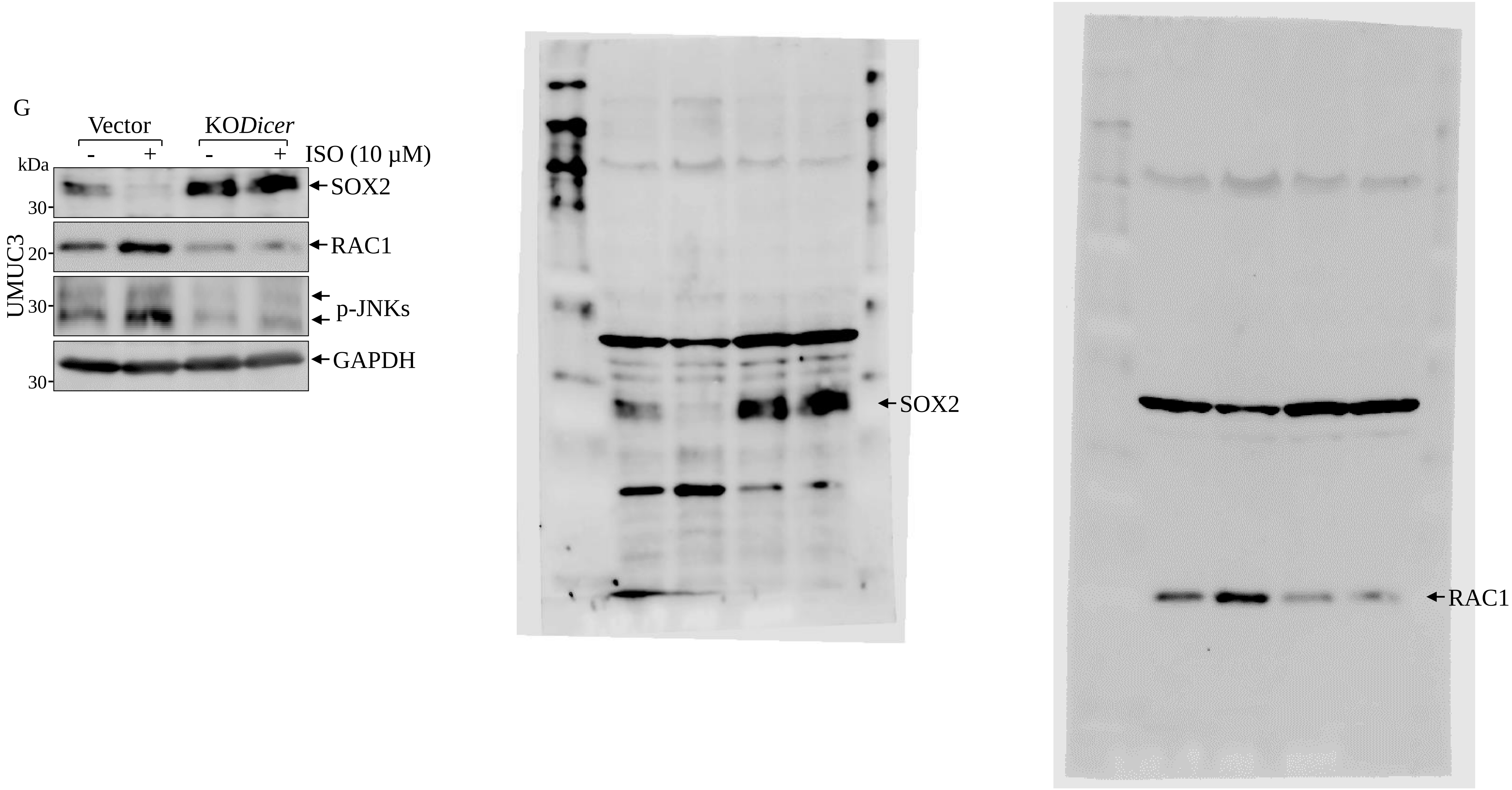
G
Vector
KODicer
ISO (10 μM)
kDa
SOX2
30
RAC1
20
UMUC3
30
p-JNKs
GAPDH
30
SOX2
RAC1

Figure 6

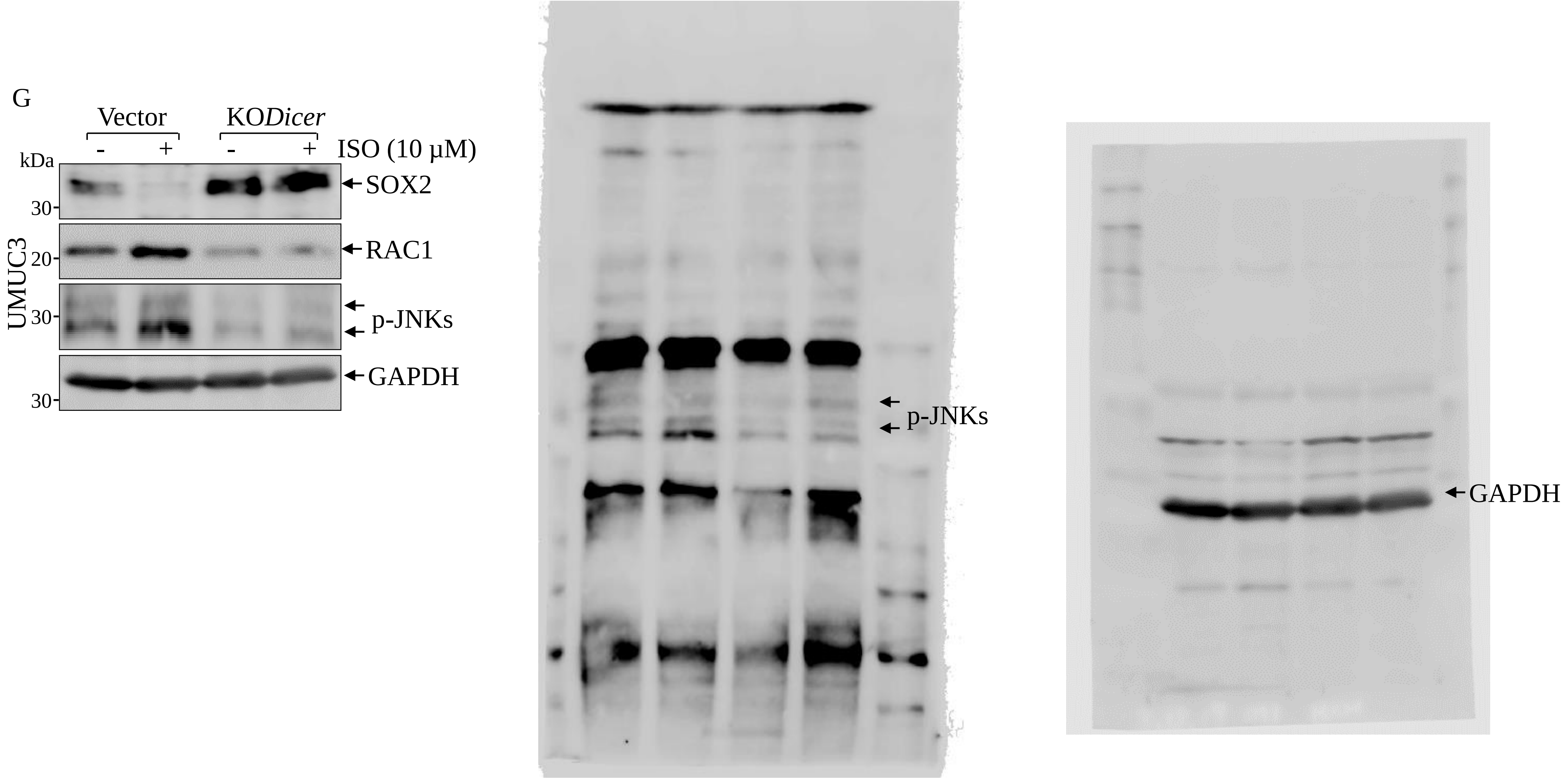
G
Vector
KODicer
- + - +
ISO (10 μM)
kDa
30
20
30
30
SOX2
RAC1
p-JNKs
GAPDH
UMUC3
p-JNKs
GAPDH

Figure 6

I

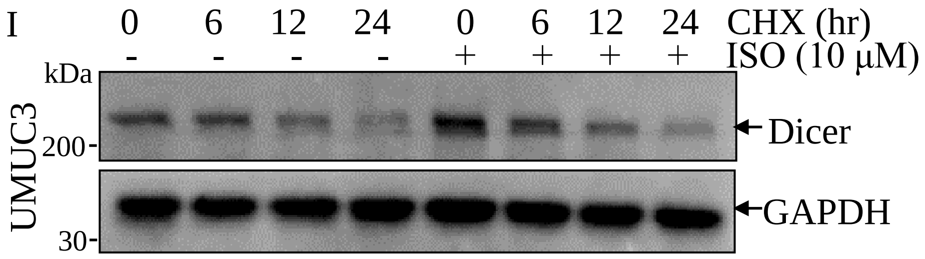
0 6 12 24 0 6 12 24 CHX (hr)
- - - - + + + + ISO (10 μM)
kDa
UMUC3
200-
30-
Dicer
GAPDH

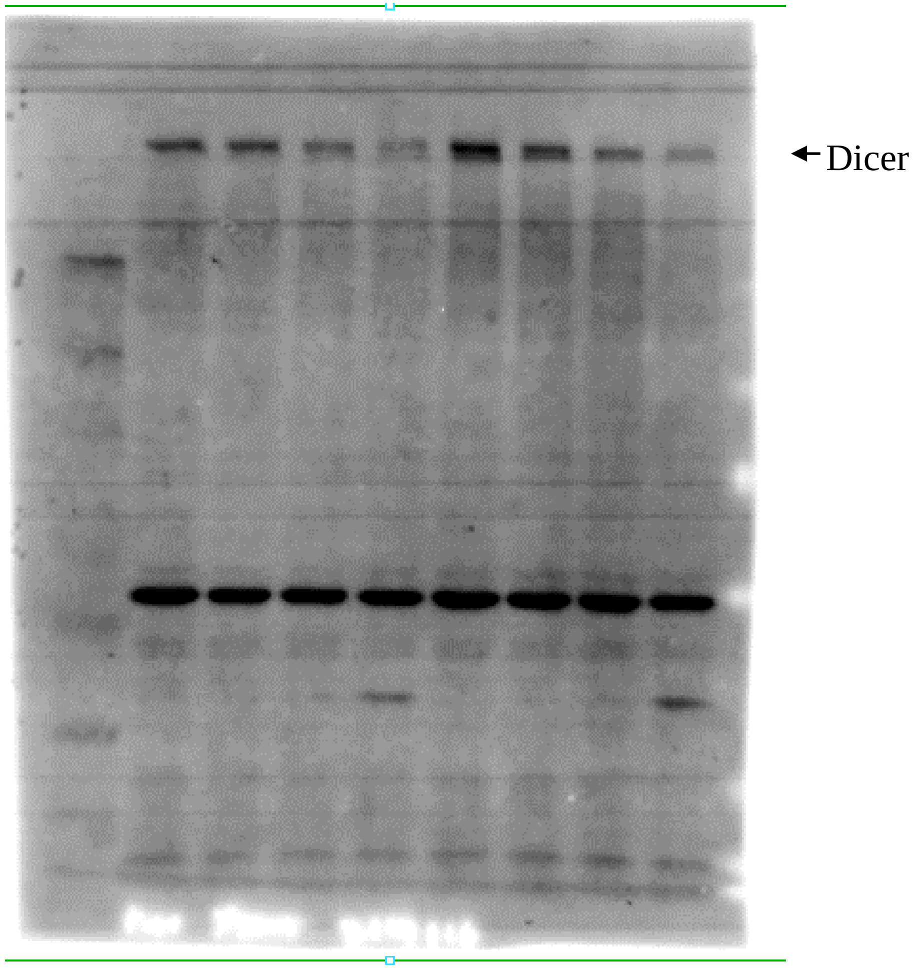
Dicer

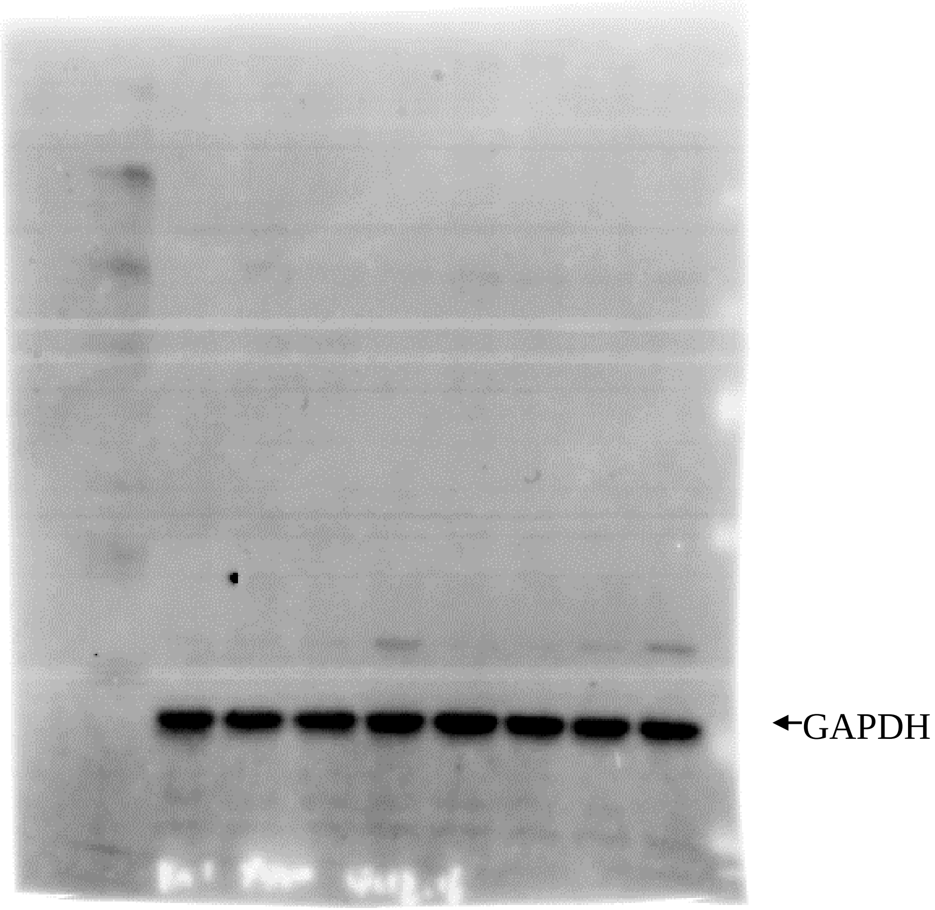
GAPDH
