# Supplementary material for: Induction of RAC1 protein translation and MKK7/JNK-dependent autophagy through dicer/miR-145/SOX2/miR-365a axis contributes to isorhapontigenin (ISO) inhibition of human bladder cancer invasion
Source: Cell Death Dis. 2022 Aug 31;13(8):753. doi: 10.1038/s41419-022-05205-w (PMC9433410; doi:10.1038/s41419-022-05205-w)
Supplement: Supplementary file 3 — author contribution [file 41419_2022_5205_MOESM3_ESM.pdf]

DECLARATION OF CONTRIBUTIONS TO ARTICLE

ADMC

Manuscript Number: CDDIS-20-0970

Journal Name: *Cell Death & Disease* (the 'Journal')

Proposed Title of the Contribution:

Induction of RAC1 Protein Translation and MKK7/JNK-Dependent Autophagy Through Dicer/miR-145/SOX2/miR-365a Axis Contributes to Isorhapontigenin (ISO) Inhibition of Human Bladder Cancer Invasion (the 'Contribution)

Author(s):

Xiaohui Hua, Daimin Xiang, Mengxin Guo, Xiaohui Qian, Ruifan Chen, Tengda Li, Zhongxian Tian, Jiheng Xu, Chao Huang, Qipeng Xie, and Chuanshu Huang* (the 'Authors')

For all *CDDis* articles, each person named as an author in the published version must be able to show he or she has contributed substantially to the article.

Authorship credit should be based on 1) substantial contributions to conception and design, acquisition of data, or analysis and interpretation of data; 2) drafting the article or revising it critically for important intellectual content; and 3) final approval of the version to be published. Authors should meet conditions 1, 2 and 3.

Any person who cannot be shown to have made a substantial contribution to the article cannot be listed as an author in the final version. The name of any person who is deemed to have made a minor contribution can, however, appear in the Acknowledgments section of the article.

Please complete the table below to indicate the contributions of all named authors to the manuscript.

Author Full Name:	Specification of Contribution to the Manuscript:
Xiaohui Hua	designed the study, detected the cells' biological function and qPCR assays, drafted the manuscript.
Daimin Xiang	designed the study and carried out the Western blot assays.
Mengxin Guo	helped to perform the bioinformatic analysis.
Xiaohui Qian	helped to perform the statistical analysis.
Ruifan Chen	helped to acquire the experimental data.
Tengda Li	helped to acquire the experimental data.
Zhongxian Tian	performed the bioinformatic analysis.
Jiheng Xu	conducted the Luciferase reporter assays.
Chao Huang	helped to acquire the experimental data.
Qipeng Xie	performed the statistical analysis.
Chuanshu Huang	designed the study and modified the manuscript.

Please complete the table below to indicate the contributions of all named authors to the figures.

Figure 1:

Chuanshu Huang, Daimin Xiang, Xiaohui Hua, Xiaohui Qian, Qipeng Xie, Chao Huang

Figure 2:

Chuanshu Huang, Daimin Xiang, Xiaohui Hua, Qipeng Xie, Chao Huang, Ruifan Chen

Figure 3:

Chuanshu Huang, Daimin Xiang, Jiheng Xu, Xiaohui Hua, Zhongxian Tian, Mengxin Guo, Qipeng Xie

Figure 4:

Chuanshu Huang, Xiaohui Hua, Daimin Xiang, Zhongxian Tian, Mengxin Guo, Qipeng Xie

Figure 5:

Chuanshu Huang, Xiaohui Hua, Daimin Xiang, Tengda Li, Chao Huang, Ruifan Chen

Figure 6:

Chuanshu Huang, Xiaohui Hua, Daimin Xiang, Jiheng Xu, Xiaohui Qian

Signed for and on behalf of the Author(s):	Print Name:	Date:
Xiaohui Hua	Xiaohui Hua	08-17-2022
